# A systematic review of observational studies on long-term air pollution exposure and epigenetic alterations in adults

**DOI:** 10.7189/jogh.16.04087

**Published:** 2026-03-20

**Authors:** Lili Yu, Yuyuan Zhao, Wenxi Chen, Guirong Yu, Mark R Miller, Xue Li, Evropi Theodoratou

**Affiliations:** 1 Centre for Global Health, Usher Institute, The University of Edinburgh, Edinburgh, Scotland, UK; 2 Department of Big Data in Health Science School of Public Health and The Second Affiliated Hospital, Zhejiang University School of Medicine, Hangzhou, China; 3 Centre for Cardiovascular Science, Queen's Medical Research Institute, The University of Edinburgh, Edinburgh, Scotland, UK; 4 Cancer Research UK Edinburgh Centre, MRC Institute of Genetics and Cancer, The University of Edinburgh, Edinburgh, Scotland, UK

## Abstract

**Background:**

Evidence suggests that environmental exposures induce epigenetic modifications that can have long-lasting effects on multiple health outcomes, and an in-depth review of the epidemiological evidence is urgent. We aimed to comprehensively assess the associations between long-term exposure to air pollution and epigenetic changes in adults.

**Methods:**

We systematically searched EMBASE, MEDLINE, and Web of Science databases for relevant articles published in English from inception through 17 November 2023. We assessed and narratively synthesised eligible studies on ambient (*i.e.* non-occupational) and epigenetic alterations in adults. We separately documented relevant occupational studies identified by the search.

**Results:**

We analysed 52 eligible articles, including 30 ambient air pollution and 22 occupational air pollution exposure studies. Long-term exposure to ambient particulate matter (PM) with aerodynamic diameters of ≤2.5 μm (PM_2.5_) and ≤10 μm, (PM_10_), and nitrogen oxides (NOx) showed no consistent association with global DNA methylation across different studies in adults. Two candidate-gene studies indicated that sex-determining region Y-box 2 (*SOX2*) hypermethylation was associated with ambient PM_2.5_ exposure. Results from epigenome-wide association studies suggest that long-term exposure to specific ambient air pollutants can alter blood methylation at up to 189 loci. In addition, decreased methylation of cg00475490 by polychlorinated biphenyls, increased methylation in cg08500171 associated with nitrogen dioxide (NO_2_) exposure, and decreased methylation in cg17629796 associated with PM_2.5_ exposure were successfully replicated in external validation cohorts. Epigenetic alterations in specific genes were associated with multiple occupational exposures.

**Conclusions:**

We demonstrated that long-term exposure to air pollution is associated with locus-specific methylation changes and histone modification in adults. Further elucidation of these epigenetic changes through epidemiological and laboratory work could provide new avenues to identify potential biomarkers linked to air pollutant exposure and to clarify their impacts on health outcomes.

**Registration:**

PROSPERO: CRD42023480771.

Air pollution is considered to be one of the foremost environmental risk factors of disability-adjusted life-years worldwide [1]. According to a 2022 World Health Organization report, it is estimated that approximately 4.2 million premature deaths globally were caused by ambient outdoor air pollution alone in 2019, predominantly from respiratory disease, heart disease, and stroke [2]. Growing evidence links long-term exposure to air pollutants to many more adverse health outcomes in most major organ systems [3]. However, the underlying biological mechanisms of air pollution-induced adverse effects throughout the body have not been fully determined.

Findings from a large body of research demonstrate that environmental exposures may induce epigenetic alterations, including DNA methylation and histone modification. Epigenetic modifications can have profound effects on gene expression and cellular function [4,5], with de novo methylation associated with tumorigenesis [6]. DNA methylation, the most common epigenetic modification, involves adding a methyl group to cytosines at cytosine-phosphate-guanine (CpG) sites and is heritable and relatively stable over time [7]. Studies have shown that several small-scale alterations in DNA caused by environmental factors might accumulate over a long period [8,9]. In addition, evidence from epigenome-wide association studies (EWAS) shows that exposure to airborne particulate matter (PM) can affect DNA methylation in blood and tissues, identifying numerous air pollution-modified CpG sites and regions [10,11]. One systematic review showed that air pollution exposure was associated with locus-specific epigenome alterations or inverse global DNA hypomethylation [12]. Nonetheless, the effects of chronic exposure to air pollution on CpG sites or regions across different population settings, as well as the latest research on this topic, have not yet been comprehensively assessed.

Histone modification affects the interaction of histone with DNA and effector proteins, thus causing modifications in the structure and function of chromatin (*i.e.* an amalgamation of DNA and protein that forms chromosome structure) [13]. Environmental contaminants have been linked to histone acetylation with global modifications, including histone H3 lysine 9 di-methylation (H3K9me2), histone H3 tri-methylated lysine 4 (H3K4me2/3), tri-methylation at lysine 27 of histone H3 (H3K27me3), and histone H3 lysine 79 trimethylation (H3K79me3) [14–17]. Histone H3 modifications are the most studied [18], and some have been reported after exposure to heavy metals [19,20] and traffic-related PM [21].

We systematically reviewed the literature on associations between epigenetic alterations and long-term air pollution exposure to consolidate the available epidemiological evidence. Specifically, we focused on ambient air pollution in the general population; however, we also identified studies assessing epigenetic changes in response to ambient air pollutants in occupational cohorts (*e.g.* PM exposure in steel workers) or air pollutants found in ambient air, but more commonly associated with occupational exposures (*e.g.* benzene exposure in leather workers). We aimed to identify fundamental biological mechanisms underlying epigenetic alterations caused by air pollution exposure, and our findings may provide useful mechanistic insights.

## METHODS

We registered the protocol in PROSPERO (CRD42023480771) on 20 November 2023 and followed the PRISMA guidelines [22].

### Literature search and selection criteria

We systematically searched Embase, Medline, and Web of Science databases from inception through 17 November 2023 using a comprehensive search strategy (Table S1 in the **Online Supplementary Document**). The search terms comprised free text and medical subject headings for epigenetic alterations and air pollutant exposures (excluding tobacco smoke exposure). We reviewed each article that included a relevant abstract and those with uncertain relevance, and downloaded them to an EndNote 20 library (Clarivate, Philadelphia, Pennsylvania, USA). By following predefined inclusion and exclusion criteria, three authors (LY, WC, and YZ) conducted a two-step parallel review of the title and abstract, and full text to determine the eligibility of the retrieved publications.

The inclusion criteria were: participants are adults (>18 years), both in the general and occupational population; long-term (>1 year) ambient and occupational exposure; epigenetic alterations as a primary or secondary outcome (including DNA methylation and histone modifications based on approaches on global DNA methylation, candidate-gene, EWAS, and histone modification); and observational studies (including longitudinal, cross-sectional, cohort, and case-control).

We excluded studies that investigated prenatal air pollution exposure effects on epigenetic alterations in children (≤18 years); were based on the elderly (>65 years); did not report the effect of air pollution on epigenetic modifications; were abstract-only publications, comments, reviews, and interviews; and were non-human studies (including research experiments conducted in animals or animal/human cell lines).

When the same study population was investigated in multiple publications, we included only the most recent (larger sample size). All included studies were written in English and published in peer-reviewed journals. We checked the reference lists of all included articles to identify any additional eligible studies.

### Data extraction

For each eligible study, two investigators (LY and WC) extracted the data independently using a predesigned data extraction form. We extracted the PubMed identification number, first author, year of publication, location, study design, number of participants, age of participants, exposure definition, type of pollutant, concentration of pollutant, exposure length, sample sites, studied region or loci, adjusted confounding factors, epigenetic alteration measurement technique, effect estimates for the major findings (coefficient of multiple linear regression or correlation coefficient with the 95% confidence intervals), and a brief summary of the reported main findings. Any discrepancies were resolved by consensus between the two data extractors. We attempted to contact the authors of the study when the exposure length for an eligible study could not be determined.

### Quality assessment

Two reviewers (LY and GY) independently assessed all included studies using the ‘Risk of Bias in Non-randomised Studies - of Exposure’ (ROBINS-E) tool. With an emphasis on environmental exposures, the ROBINS-E tool is adapted from the original Risk of Bias in Non-randomised Studies of Interventions tool [23]. We evaluated seven main methodological aspects (*i.e.* domains) deduced from ROBINS-E, including bias due to confounding, bias due to exposure classification, bias due to missing data, bias in outcome measurement, bias due to departures from intended exposures, bias in the selection of reported results, and bias in selecting participants in the study, with the risk of bias judgments expressed as either ‘low’, ‘some’, or ‘high’ (Table S2 in the **Online Supplementary Document**). Any disagreements were resolved by consensus between the reviewers. We recorded the overall risk of bias for each study as the highest risk across any domain.

## RESULTS

### Literature review

**Figure 1:**
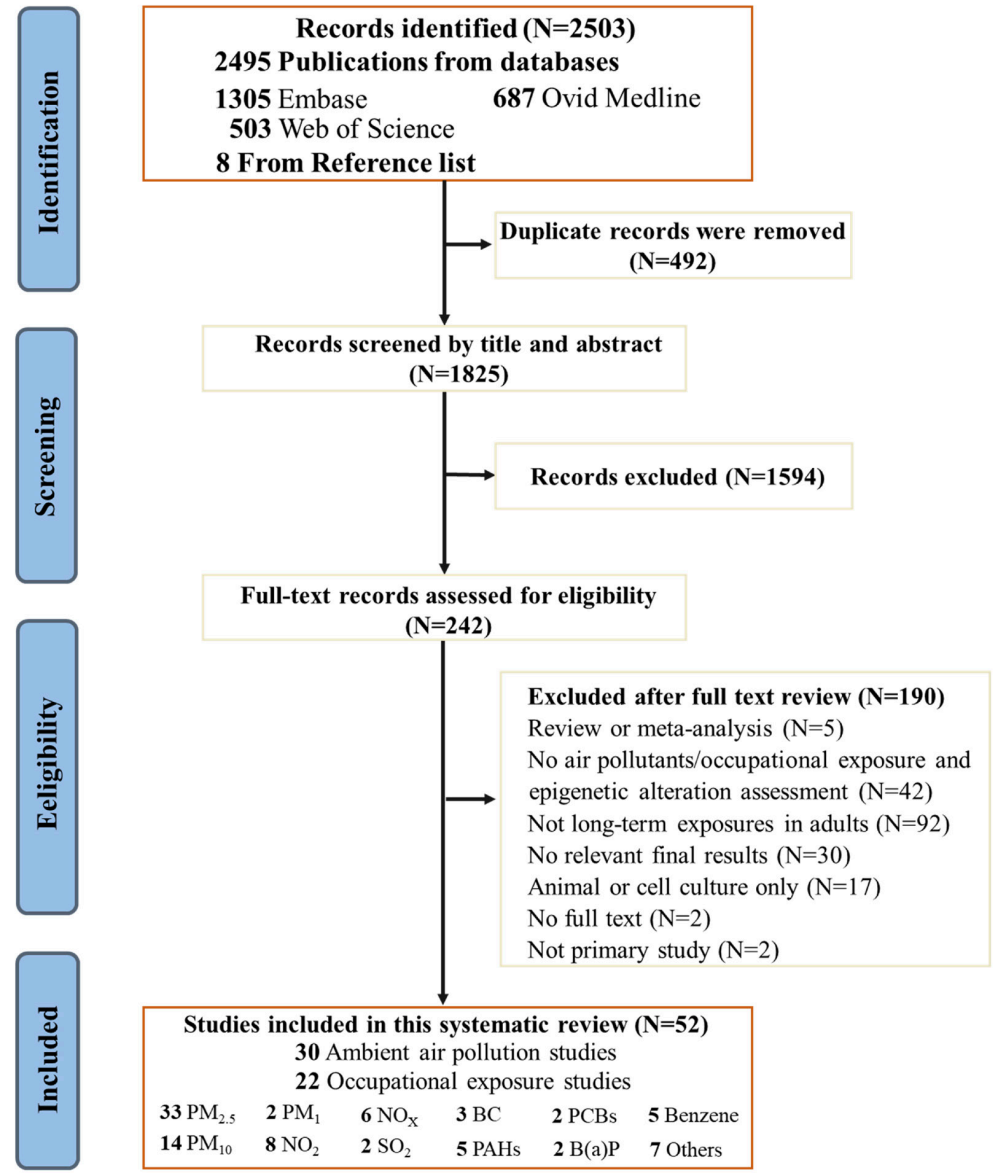
Flowchart of literature search. BC – black carbon, NO_2_ – nitrogen dioxide, NOx – nitrogen oxides, PAH – polycyclic aromatic hydrocarbon, PCB – polychlorinated biphenyl, PM – particulate matter, SO_2_ – sulfur dioxide.

We identified 2495 publications across Embase, Medline, and Web of Science databases (**Figure 1**). After removing 492 duplicates, we screened 1825 records by title and abstract, and selected the remaining 242 articles for full-text screening. Finally, 52 publications met the inclusion criteria, with 44 retrieved from the above three databases [17,24–65] and eight additional articles identified from the reference lists of other papers [66–73].

### Study characteristics

There were six cohort [26,37,45,54,69,73], three case-control [27,34,56], and 43 cross-sectional studies [17,24,25,28–33,35,36,38–43,45–52,54,55,57–68,70–72] (**Tables 1–3**; Tables S3–5 in the **Online Supplementary Document**). Overall, 30 studies were classed as ‘ambient air pollution exposure’, with a median exposure window of three years (range = 1–29.6) [17,27,29–35,37,38,40,47,48,50,52–56,58–61,63,67,70,71], and 22 classed as ‘occupational exposure’, with a median of five years (range = 1–31) [17,24,25,35,40–45,49,52,57,62,64–66,68,69,72,73]. Some studies measured exposure to air pollutants using land-use regression models based on data from stationary monitoring networks [6,30,31,33,35,50,51,60,61]. Other studies used cyclone- or battery-operated channel optical particle counters as personal air pollution monitors during working hours at workplaces [17,25,40–42,44,52,56,57,62,68,69,73]. In particular, PM with aerodynamic diameters of ≤2.5 μm (PM_2.5_) and ≤10 μm (PM_10_), nitrogen oxides (NOx), nitrogen dioxide (NO_2_), polycyclic aromatic hydrocarbons (PAHs), solid fuel, benzo(a)pyrene (B(a)P), polychlorinated biphenyls (PCBs), sulphur dioxide (SO_2_), and bisphenol A (BPA) were analysed in ambient air pollution studies, whereas PM_1_, PM_2.5_, PM_10_, benzene, black carbon, B(a)P, PAHs, and formaldehyde were evaluated in occupational settings. PM (including PM_1_, PM_2.5_, PM_10_) was most frequently reported (n = 34) [17,26–34,36–44,47,48,50–52,54,55,57–61,71,74], followed by NO_2_ (n = 8) [6,32,34,39,50,51,59,60], NO_X_ (n = 6) [30–32,34,50,51], PAHs (n = 5) [24,38,46,52,65,66], benzene (n = 5) [45,68,69,72,73], black carbon (n = 3) [35,49,59], B(a)P (n = 2) [39,64], PCBs (n = 2) [67,70], SO_2_ (n = 2) [32,59], BPA (n = 1) [53], and formaldehyde (n = 1) [25]. Most studies used blood samples to extract DNA (n = 48) [17,24,25,28,30–52,54–73], whereas brain and breast tissue samples were used in two studies [26,27] and semen samples in two studies [29,53]. Of these, studies determined the occupational exposure by analysing biomarkers in the blood [43] or urine [24,64–66]. Eleven epigenetic alteration assessment techniques were applied, including pyrosequencing-based, array-based, high-throughput sequencing-based, analytical chemistry-based, and antibody-based enrichment approaches (Table S6 in the **Online Supplementary Document**), with Pyrosequencing [24,27,28,38,40,41,43,44,49,52,57,58,62,65,66,68,69] and Illumina Infinium Human Methylation 450K BeadChip Array [6,30,31,33,34,36,48,50,51,58,60,61] methods being the most used to estimate ambient air pollution-induced DNA methylation.

**Table 1:** Characteristics and summary of studies using a global methylation approach

Author	Year	Country	Study population	Study design	Sample size	Age in years, x̄ (SD)	Pollutant type	Studied region or loci	Main findings
Cheng et al. [29]	2022	China	NMU-LIFE study of the general population	Cross-sectional	1554	Range: 20–45; normal semen quality group: 30.6; abnormal semen quality group: 31.4	PM_10_	Global DNA Methylation: (5 mC) and Global DNA Hydroxymethylation (5-hmC)	PM_10_ exposure positively associated with 5-hmC levels (*β* = 0.002; per μg/m^3^)
Chi *et al.* [30]	2016	USA	MESA in the general population	Cross-sectional	1207	69.6 (9.4)	PM_2.5_, NO_X_	Global DNA Methylation: Alu and LINE-1	Median DNA methylation of Alu and LINE-1 was not associated with PM_2.5_ (Alu, *β* = −0.003; 95% CI = −0.006, 0.001; LINE-1, *β* = −0.003; 95% CI = −0.007, 0.001; per 2.5 μg/m^3^) or NOx (Alu, *β* = −0.001; 95% CI = −0.006, 0.008; LINE-1, *β* = −0.0004; 95% CI = −0.009, 0.008; per 30 ppb)
Goobie *et al.* [37]	2023	USA	The University of Pittsburgh Simmons Centre for ILD Registry (Simmons) or the US-wide PFF Patient Registry	Cohort	1059	Simmons cohort: 68 (61–74); PFF cohort: 69 (64–74)*	PM_2.5_	Global DNA methylation	Higher PM_2.5_ (1-y period exposures prior to blood collection) were associated with higher global DNA methylation percentage (%5 mC) in both Simmons (*β* = 0.03; 95% CI = −0.004, 0.06; per 11.2 μg/m^3^) and PFF (*β* = 0.03; 95% CI = −0.007, 0.06; per 7.6 μg/m^3^) cohorts
Plusquin *et al.* [11]	2017	Italy, Netherlands	Italian and Dutch components of the EPIC study of the general population	Cross-sectional	613	Italy: 54.2 (7.1); Netherlands: 58.8 (5.6)	NO_X_, NO_2_, PM_10_, PM_2.5_	Global DNA methylation	NO_2_ (EPIC-Italy *β* = −0.00007; SE = 0.00003; EPIC-NL *β* = −0.00388; se = 0.00133; per μg/m^3^) and NOx (EPIC-Italy *β* = −0.00001; SE = 0.00006; per μg/m^3^) were inversely associated with global DNA hypomethylation in EPIC-Italy cohort, but not for PM_2.5_ and PM_10_
Tao *et al.* [56]	2014	Poland	Residence of non-smoking women in Warsaw	Case-control	42	59.4 (79.9)	Solid fuel	Global DNA methylation	The levels of LUMA methylation for women who had ever exposed to both coal and wood were reduced compared to women without exposure (6.70%; 95% CI = −13.36, −0.04)
Wang *et al.* [58]	2020	USA	Selected from the Sister Study	Cross-sectional	1373	Sub-cohort1:55.8 (8.9); sub-cohort2: 56.7 (8.8)	PM_2.5_	Global DNA methylation: LINE-1	There is no association between PM_2.5_ and LINE-1
Xu *et al.* [61]	2023	Australia	AMDTSS female participants	Cross-sectional	479	56.4 (7.9)	PM_2.5_	Global DNA methylation: Alu, LINE-1, LTR	The 3-y average wildfire-related PM_2.5_ was negatively, but not significantly (*P* > 0.05) associated with all seven global DNA methylation measures
Yadav *et al.* [63]	2021	India	A major research project of the general population	Cross-sectional	512	Low polluted: 47.9 (8.9); high polluted: 48.2 (10.2)	Total air pollution	Global DNA methylation	Air pollution showed a significant effect on global DNA methylation only among individuals with hyperhomocysteinemia (*β* = −0.936), not among those with normal homocysteine levels (*β* = 0.23)

ALU – Arthrobacter luteus, AMDTSS – Australian Mammographic Density Twins and Sisters Study, CI – confidence interval, LINE-1 – long interspersed nuclear elements-1, LTR – long terminal repeat, MESA – Multi-Ethnic Study of Atherosclerosis, NMU-LIFE – Nanjing Medical University Longitudinal Investigation of Fertility and the Environment, NO_2_ – nitrogen dioxide, NOx – nitrogen oxides, PFF – Pulmonary Fibrosis Foundation, PM – particulate matter, SD – standard deviation, x̄ – mean

*Values presented as median (interquartile range).

**Table 2:** Characteristics and summary of studies using a candidate-gene methylation approach

Author	Year	Country	Study population	Study design	Sample size	Age in years, x̄ (SD)	Pollutant type	Studied region or loci	Main findings	
Callahan *et al.* [27]	2018	USA	WEB Study	Case-control	1170	35–79*	TSP	Gene-based: *SFN*, *SCGB3A1*, *RARB, GSTP1*, *CDKN2A CCND2*, *BRCA1*, *FHIT*, and *SYK*	Ambient air pollution was significantly associated with DNA methylation of the tumour suppressor genes SCGB3A1, SYK, and CCND2 in breast tumour tissue
Cantone *et al.* [28]	2020	Italy	ED of Fondazione IRCCS Ca’ Granda, Ospedale Maggiore Policlinico, with a diagnosis of acute ischemic stroke	Cross-sectional	55	74.6 (13.9)	PM_2.5_	Gene-based: *ARNTL*, *CLOCK*, *CRY2*, *PER1*, *PER2*, *CRY1*, *NPAS2*	PM_2.5_ exposure was not associated with *ARNTL*, *CLOCK*, *NPAS2*, *CRY2*, *PER1*, *PER2*, *CRY1,* and *PER3* methylation (*P* > 0.05)
Chou *et al.* [32]	2020	China	TWB database of the general population	Cross-sectional	496	No regular exercise: 45.832 (0.635), regular exercise: 55.185 (0.660)	PM_2.5_	Gene-based: *DLEC1* promoter	PM_2.5_ was positively associated with *DLEC1* methylation.
Song *et al.* [53]	2019	China	A male prospective cohort study in China	Cohort	157	>29*	BPA	Gene-based: *ACHE*	Urine BPA concentration positively associated with 5hmC rate of the sperm *ACHE* gene
Tantoh *et al.* [54]	2019	China	TWB data set of the general population	Cross-sectional	708	Men: 49.42 (11.76); women: 49.49 (10.97)	PM_2.5_	Gene-based: *AHRR*	A unit increase in PM_2.5_ significantly associated with lower cg05575921 (*AHRR*) methylation levels (*β* = −0.00115; per μg/m^3^)
Tantoh *et al.* [55]	2019	China	TWB data set of the general population	Cross-sectional	461	Men: 48.86 (11.74); women: 48.78 (11.01)	PM_2.5_	Gene-based: *SOX2*	x̄ PM_2.5_ level (2006–2011) associated with higher *SOX2* promoter hypermethylation level
Su *et al.* [71]	2020	China	Taiwanese adults aged 30–70 who have no personal history of cancer	Cross-sectional	948	No exercise: 46.1 (0.46); exercise:54.4 (0.5)	PM_2.5_	Gene-based: *SOX2*	PM_2.5_ significantly associated with higher levels of *SOX2*-promoter hypermethylation
Wang *et al.* [59]	2020	USA	Selected from the Sister Study	Cross-sectional	1373	Sub-cohort1: 55.8 (8.9); subcohort2: 56.7 (8.8)	PM_2.5_	Gene-based: *TNF-α, TLR-2*	Estimated change of −6.5% (95% CI = −13.34%, 0.35%) in x̄ methylation of TNF-α per 5 μg/m^3^ increase in PM_2.5_

BPA – bisphenol A, CI – confidence interval, ED – emergency department, NO_2_ – nitrogen dioxide, NOx – nitrogen oxides, PM – particulate matter, SD – standard deviation, TSP – total suspended particulates, TWB – Taiwan Biobank, WEB – Western New York Exposures and Breast Cancer, x̄ – mean, 5hmC – 5-hydroxymethylcytosine

*Values presented as a range.

**Table 3:** Characteristics and summary of studies using an EWAS approach*

Author	Year	Country	Study population	Study design	Sample size	Age in years, x (SD)	Pollutant type	Main findings
Chi *et al.* [30]	2016	USA	MESA in the general population	Cross-sectional	1207	69.6 (9.4)	PM_2.5_, NO_X_	Long-term PM_2.5_ exposure was significantly associated with 5 candidate CpGs methylation, including cg20455854 (*β* = 0.139; 95% CI = 0.074, 0.203), cg07855639 (*β* = 0.081; 95% CI = 0.043, 0.120), cg07598385 (*β* = 0.108; 95% CI = 0.056, 0.160), cg17360854 (*β* = 0.081; 95% CI = 0.042, 0.120), and cg23599683 (*β* = −0.057; 95% CI = −0.085, −0.029) (all per 2.5 μg/m^3^) but not NOx
Chi *et al.* [31]	2022	USA	MESA in the general population	Cross-sectional	1207	69.6 (9.4)	PM_2.5_, NO_X_	Long-term PM_2.5_ exposure was significantly associated with 3 methylated CpGs, and NOx with cg11756214 within *ZNF347*
de F C Lichtenfels *et al.* [6]	2018	Netherland	Lifeline cohort study participants of the general population	Cross-sectional	1017	47.3 (11.0)	NO_2_, PM_2.5_, PM_10_	7 CpG sites were significantly associated with NO_2_ levels; no associations were found for PM_10_ and PM_2.5_ exposure at genome-wide level (*P* < 1.19 × 10^−7^)
Eze *et al.* [33]	2020	Switzerland	The SAPALDIA cohort of the general population	Cross-sectional	1389	SAPALDIA2: 50 (18), SAPALDIA3: 58 (18)	NO_2_, PM_2.5_	Reported top 10 pm_2.5_ and NO_2_-related CpGs, but not at the epigenome-wide significance level (*P* < 1.16 × 10^−7^)
Fiorito *et al.* [34]	2018	Italy	EPIC-Italy study of the general population	Case-control	320	CCVD: 54.8 (7.4); control: 54.9 (7.1)	NO_2_, NOx, PM_2.5_	Identified enrichment of altered DNA methylation in ‘ROS/Glutathione/ Cytotoxic granules’ and ‘Cytokine signalling’ pathways related genes associated with NO_2_, NOx, PM_2.5_, respectively.
Gondalia *et al.* [36]	2019	USA	Participants of 2 cohorts (WHI, ARIC)	Cross-sectional	8397	61.3 (7.4)	PM_10_, PM_2.5_	55 suggestively significant PM-associated CpG methylation were identified from trans-ethnic, fixed-effects inverse-variance-weighted meta-analyses
Honkova *et al.* [39]	2022	Czech Republic	Nonsmoking city policemen from 3 cities of Czech Republic	Cross-sectional	125	Prague: 40.4 (9.3); Ostrava: 39.3 (9.2); Ceske Budejovice: 38.0 (6.4)	PM_2.5_, B(a)P, NO_2_	13 643 DML were identified from a comparison between the Prague and Ostrava groups
Liu *et al.* [46]	2021	China	3 panels of the healthy Chinese population (WHZH panel, COW panel, and Shiyan panel)	Cross-sectional	304	WHZH-Wuhan: 51.3 (13.9); WHZH-Zhuhai: 61.7 (11.7); SY: 40.0 (10.9); COW: 44.9 (8.4)	PAHs	Methylation level of cg09235308 was positively associated with urinary total OH-PAHs, but no significant epigenome-wide associations for the other 9 urinary OH-PAHs
Messingschlager *et al.* [47]	2023	Germany	General participants from Simmerath (Low PM_10_) and Stuttgart (Moderate PM_10_)	Cross-sectional	60	Stuttgart: 28 (20–36); Simmerath: 27 (20–39)†	PM_10_	231 DMRs between moderately and lowly PM_10_-exposed individuals
Mu *et al.* [48]	2023	China	Wuhan-Zhuhai cohort of the general population	Cross-sectional	402	58.4 (7.89)	PM_2.5_	10 pm_2.5_-related CpG sites (mapped to 7 different genes) were observed
Plusquin *et al.* [11]	2017	Italy, Netherlands	Italian and Dutch components of the EPIC study of the general population	Cross-sectional	613	EPIC-Italy: 54.2 (7.1); EPIC-Netherlands: 58.8 (5.6)	NOx, NO_2_, PM_10_, PM_2.5_	NO_2_ and NOx were inversely associated with global DNA methylation, but not with PM_2.5_ and PM_10_
Sayols-Baixeras *et al.* [51]	2019	Italy, Spain	General-based individuals from the REGICOR cohort and EPIC-Italy study	Cross-sectional	1084	REGICOR: 63.3 (11.7); EPIC-Italy: 54.2 (7.1)	PM_10_, PM_2.5_, NOx, NO_2_	Did not identify any new genomic loci associated with long-term air pollution and did not replicate any previously identified loci
White *et al.* [60]	2019	USA	The Sister Study cohort of women participants who had a sister with breast cancer but no history of breast cancer themselves	Cross-sectional	2747	45–65†	PM_2.5_, PM_10_, NO_2_	NO_2_ was associated with methylation at 2 CpG sites
Wang *et al.* [59]	2020	China	Chinese Han general population-based cohort	Cross-sectional	120	HPR: 40.6 (2.2); LPR: 40.7 (3.5)	PM_2.5_, PM_10_, SO_2_, NO_2_, CO	Genome-wide methylation analysis revealed 191 hypomethylated and 180 hypermethylated DMRs (*P* < 0.1) in subjects from the two areas, and these DMRs were located primarily in gene regulatory elements such as promoters and enhancers
Xu *et al.* [61]	2023	Australian	Female participants in the AMDTSS	Cross-sectional	479	56.4 (7.9)	PM_2.5_	26 CpGs and 33 DMRs associated with wildfire-related PM_2.5_ (*P* < 0.05) mapped to 47 genes enriched for pathways related to inflammatory regulation and platelet activation
Curtis *et al.* [67]	2021	USA	Those with the highest exposure to PBB selected from the Michigan PBB Registry	Cross-sectional	641	54.3 (12.8)	PCBs	Current total PCBs level associated with the methylation proportion at 1345 CpGs
Pittman *et al.* [70]	2020	USA	Selected from the Anniston Community Health Survey	Cross-sectional	817	57.6 (4.02)	PCBs	28 significant CpGs were associated with PCBs, representing 17 unique genes

AMDTSS – Australian Mammographic Density Twins and Sisters Study, B(a)P – benzo(a)pyrene, CCVD – cardio- and cerebrovascular disease, CI – confidence interval, CO – carbon monoxide, CpG – cytosine-guanine dinucleotide, DML – differentially methylated CpG loci, DMR – differentially methylated region, EWAS – epigenome-wide association Study, HPR – highly polluted region, LPR – less polluted region, MESA – Multi-Ethnic Study of Atherosclerosis, NO_2_ – nitrogen dioxide, NOx – nitrogen oxides, PAH – polycyclic aromatic hydrocarbon, PBB – polybrominated biphenyl, PCB – polychlorinated biphenyl, PM – particulate matter, SD – standard deviation, SO_2_ – sulphur dioxide, x̄ – mean

*All studies used an epigenome-wide approach.

†Values presented as median (interquartile range) or as a range.

### Risk of bias assessment

The domains with the highest risk of bias were confounding, exposure classification, missing data, outcome measurement, and exposure measurement (Table S7 in the **Online Supplementary Document**).

For confounding, we considered socioeconomic status, race and ethnicity, seasons, temperature, and smoking status to be the largest threats of bias. For studies where adjusted estimates were available (*e.g.* age, race/ethnicity, sex, study site, income, education, seasons, temperature, and smoking status), the bias was likely to be low [29-31,33,36,61], but when only crude estimates were available, we considered the study at high risk of bias [26,39,47,52,64]. For exposure classification, we considered studies that explored long-term exposure (>1 year) using models with good performance and/or estimated air pollution exposure using monitoring stations at low risk of bias [4,17,24,26,27,29-34,36-42,47–51,54,55,58–61,71,73]. We considered high risk when exposure levels were reported for long-term exposures using a model with poor performance or unclear exposure estimation [35]. For missing data, we considered studies that did not mention follow-up in cohort studies to be at high risk of bias [26,37,69,73]. For outcome measurement, studies with explicit methodologies for sample collection and technique controls in examining epigenetic alterations were considered low risk, whereas studies were at some risk of bias in assessing epigenetic alterations [24,25,27,35,37–39,41–45,49,52,53,56,63–69]. For the selection of reported results, studies reporting *P*-values with multiple test corrections (*e.g.* false discovery rate) were considered at low risk of bias; otherwise, at some risk of bias [17,28,40,42,43,45]. Regarding participant selection, the risk of bias for each study was assessed as low (Table S2 in the **Online Supplementary Document**). The overall risk of bias was considered ‘low’ for four studies [30,31,33,61], ‘some’ for 25 studies [17,24,27–29,32,34,36,46,48,50,51,54–60,63,65–67,70,71], and ‘high’ for 23 studies [6,25,26,34,37–45,47,49,52,53,62,64,68,69,72,73] (Figure S1 in the **Online Supplementary Document**).

### Ambient air pollution studies

Overall, 30 studies measured exposure to long-term ambient air pollutants and epigenetic alterations [27-34,36,37,39,46–48,50,51,53–56,58–61,63,67,70,71]. Global DNA methylation approaches were reported in eight studies [29,30,37,50,56,58,61,63], candidate-gene approach in eight studies [27,28,32,54–56,59,72], the EWAS approach in 17 studies [6,30,31,34,35,37,40,47,48,50,51,59–61,67,70], and the histone modification approach in one study [26]. Among them, four studies used ≥2 approaches [30,50,58,61].

#### Global DNA methylation studies

We analysed eight studies [29,30,37,50,56,58,61,63] that assessed the association between ambient long-term exposure to specific air pollutants and global DNA methylation patterns (**Table 1**; Table S5 in the **Online Supplementary Document**). Seven studies reported a sample size of >100 participants [29,30,36,50,58,61,63]. There were seven global DNA methylation studies investigating DNA methylation levels in blood samples [30,37,50,56,58,61,63], and one in semen samples [29]. Three studies used methylation measurements of repetitive elements/regions (LINE-1 and Alu) as proxies for global DNA methylation levels [30,58,60]. Most of these studies (n = 7) [29,30,37,50,58,59,61] investigated changes in global DNA methylation following increased exposures to ambient PM_2.5_ or PM_10_. Among the ambient PM (PM_2.5_ or PM_10_) exposures reported in the included studies, two studies found that PM exposure was significantly associated with higher levels of the alternative epigenetic DNA modifications 5-methylcytosine and 5-hydroxymethylcytosine in blood and semen, respectively [29,37]. Nevertheless, four studies found no statistically significant association between long-term exposure to PM_2.5_ and PM_10_ and blood global DNA methylation [30,50,58,61].

Studies investigating exposure to NO_2_ (n = 1) [50], NO_X_ (n = 2) [30,50], solid fuel (n = 1) [56], and total air pollution (n = 1) [63] were also identified. Discrepant findings were found between studies exploring the association between global DNA methylation and exposure to ambient NOx. One study [50] showed an inverse association between NOx exposure and global DNA hypomethylation, whereas another study [30] suggested no significant association between NOx exposure and median DNA methylation of LINE-1.

#### Candidate-gene methylation studies

We identified eight studies investigating the association between air pollution and candidate-gene methylation (**Table 2**; Table S5 in the **Online Supplementary Document**), six of which assessed blood samples [28,32,54,55,58,71], whereas one in the breast tumour tissue sample [27] and one in the semen sample [53]. Air pollutants in these candidate-gene studies included PM (n = 6) [27,28,32,54,55,58,71] and BPA (n = 1) [53].

PM_2.5_ exposure was significantly and positively associated with higher levels of promoter methylation of blood sex-determining region Y-box 2 (*SOX2*) in two studies [55,71]. Long-term ambient PM_2.5_ exposure was significantly associated with decreased methylation of the deleted in lung and oesophageal cancer 1 (*DLEC1*) promoter and cg05575921 within the aryl hydrocarbon receptor repressor (*AHRR*) [32,54]. Two studies reported gene methylation in breast and sperm samples [27,53]. Total suspended ambient particulates were suggested to be significantly associated with DNA methylation of tumour suppressor genes (*SCGB3A1*, *SYK*, and *CCND2*) in breast tumour tissue [27]. Urine BPA concentration showed a positive association with sperm *ACHE* hydroxymethylation in men [53] (Table S8 in the **Online Supplementary Document**).

#### EWAS studies

Regarding the 17 EWAS studies, 16 were cross-sectional studies [6,30,31,33,36,39,46–48,50,51,59–61,67,70], and one was a case-control study [34] (**Table 3**; Table S9 in the **Online Supplementary Document**). Samples of >100 participants were analysed in the discovery phase of 16 studies [6,30,31,33,34,36,39,46,48,50,51,59–61]. All identified EWAS studies used blood samples, and the Infinium Human Methylation 450K BeadChip Array (Illumina) technique was the most commonly applied to assess the air pollution-induced CpG sites or regions [6,30,31,33,34,36,46,50,51,60,61], followed by Illumina Infinium 850K MethylationEPIC BeadChip [39,48,67,70], and whole-genome bisulfide sequencing [47,59]. Ambient PM exposure was assessed in 14 EWAS studies [6,30,31,33–35,39,47,48,50,51,59–61], NOx and NO_2_ in ten studies [6,30,31,33,34,39,50,51,59,60], PCBs in two studies [67,70], and PAHs in one study [48].

All the EWAS studies indicated that long-term exposure to ambient air pollution was associated with alterations in blood methylation of up to 189 unique loci (Table S9 in the **Online Supplementary Document**). The largest and most recent study, conducted by Eze and colleagues [33], included 1389 individuals and reported the top ten ambient NO_2_-associated and ten ambient PM_2.5_-associated CpGs in whole blood. One epigenome-wide study [46] found that the methylation level of cg09235308 in plectin 1 was positively associated with ten urinary mono-hydroxylated PAHs. In addition, the methylation in pescadillo ribosomal biogenesis factor 1 (*PES1*) was reported to be associated with both ambient PM_2.5_ and PM_10_ exposure in a multicentre prospective cohort [50]. Wang and colleagues [59] applied a multi-exposure model that included PM_2.5_, PM_10_, SO_2_, NO_2_, and CO, and reported that combined exposure to long-term ambient air pollution led to 371 differentially methylated regions (DMRs), located primarily in gene regulatory elements such as promoters and enhancers.

Four EWAS studies found significant signals in a discovery population and validated successfully in the external replication populations [6,50,51,70]. Pittman and colleagues [70] reported significant associations between exposures to PCBs and the methylation of 28 CpGs in whole blood in the discovery cohort. However, only one association was statistically significant in the replication external cohort: cg00475490 in the gene of serine protease 23 (*PRSS23*). Additionally, Eze and colleagues [33] replicated two previously identified air pollution-associated EWAS signals; increased methylation in cg08500171 in the gene of branched-chain-amino-acid transaminase 2 (*BAT2*) related to NO_2_ exposure and decreased methylation in cg17629796 related to PM_2.5_ exposure (Table S10 in the **Online Supplementary Document**). These three EWAS signals highlight strong evidence of the association between air pollutants and DNA methylation.

#### Histone modification studies

Ambient air pollution exposure and histone modification were assessed in one study [26]. It reported an inverse association between histone modification (mainly H3K9me2) and exposure to ambient PM_2.5_ in brain samples [26] (Tables S4 and S5 in the **Online Supplementary Document**).

### Findings in occupational air pollution exposure studies

We identified 22 studies [17,24,25,35,38,40–45,49,52,57,62,64–66,68,69,72,73] that assessed the association between long-term occupational (including brick makers, boilermakers, shoe makers, electronic-waste recyclers, truck drivers, traffic policemen, welders, salon, steel, blue-collar, tanneries and coke oven plant workers) air pollution exposures and global DNA methylation patterns (n = 7) [24,25,35,43,44,49,65], candidate-gene studies (n = 13) [38,40–42,52,57,62,64,66,68,69,72,73], and histone modifications (n = 2) [17,45] (Tables S3–5 in the **Online Supplementary Document**).

Two studies found no statistically significant association between long-term occupational air pollution exposure (electronic-waste recyclers and boilermakers) and LINE-1/Alu methylation for PM_2.5_ and PM_10_ [43,44]. In candidate gene studies, occupation-related genes with epigenetic alterations were reported in our study. One study showed that methylation levels of adenomatous polyposis coli (*APC*) were positively associated with occupational exposure to PM_10_ or PM_1_ in steel plant workers, whereas tumour protein P53 (*p53*) and ras association domain family 1 isoform A (*RASSF1A*) were not [40]. Another study [57] reported that PM_10_ or PM_1_ was inversely associated with methylation of nitric oxide synthase 3 (*NOS3*) in steel workers, whereas methylation of endothelin 1 was not. Methylation of multiple tumour suppressor 1 (*p16*) was explored in two studies [40,72], with one [40] finding that methylation status in *p16* in steel workers was not significantly associated with PM_1_ or PM_10_, while the other [72] indicated no association between the *p16* gene methylation and benzene exposure in paint, shoe and printing workers. Two individual studies were conducted for ambient PAHs exposure, one found that 1-hydroxypyrene urine concentration was negatively associated with DNA hypomethylation of the interleukin 12 [24], and the other observed PAH metabolite concentrations were related to hypomethylation of coagulation factor II (thrombin) receptor-like 3 (*F2RL3*) and *AHRR* in chimney sweep workers [66]. Occupational exposure to PM_1_ and PM_10_ in steel workers was not associated with blood histone modification of the genes H3K4me2 and H3K9ac [17] (Tables S3, S4, and S8 in the **Online Supplementary Document**)_._

## DISCUSSION

In this systematic review, we assessed the evidence for associations between long-term exposure to ambient and occupational air pollution with epigenetic modification in adults. More studies evaluated the association between air pollution exposure and locus-specific DNA methylation than those evaluating histone modifications. The studies identified showed high heterogeneity in exposure modelling and epigenetic alteration assessment. While most studies investigated ambient PM or NOx exposure, there was no consistent evidence of an association with global DNA methylation. Among candidate-gene studies, hypermethylation of the *SOX2* gene was positively associated with ambient PM_2.5_ exposure, although this association was based on only two studies. There were 17 EWAS studies that found that long-term exposure to ambient air pollution was associated with methylation changes at up to 189 unique loci in blood. In EWAS studies successfully replicated in external validation cohorts, methylation was altered at loci such as cg00475490 (*PRSS23*) to PCBs, cg08500171 (*BAT2*) to NO_2_, and cg17629796 to PM_2.5_ exposure. In PM exposure in occupational settings, there was no significant effect on global DNA methylation. Although a greater number of potential associations were not significantly altered, PM exposure in steel workers was found to modify the methylation status of specific candidate genes. There was no change for histone modification of H3K genes.

DNA methylation patterns are disrupted in human malignant tumours and cell types, with genome-wide hypomethylation and gene-specific hypermethylation occurring simultaneously in the same cell, leading to gene expression and chromosome instability owing to large-scale alterations [75]. In addition, inhaled air pollutants are associated with adverse effects across many organ systems in adults via epigenetic changes [76,77]. While many air pollutants are harmful to health, associations with ambient PM exposure tend to be stronger and more consistent [78]. We found that PM_2.5_ and PM_10_ were the most investigated air pollutants in global DNA methylation studies. However, we observed discrepant findings regarding associations between PM_2.5_ and PM_10_ exposure and DNA methylation levels. Two studies reported a positive association between exposure to ambient PM_2.5_ or PM_10_ and global DNA methylation [29,37], while most studies reported no significant associations between these air pollutants and global DNA methylation [30,50,58,61]. We also found an inconsistency between ambient NOx exposure and global DNA methylation [30,50]. One potential explanation is that measurement error in DNA methylation (*i.e.* batch, chip, or cell composition effects) may result in differences in epigenetic alteration levels, thereby diminishing the statistical power required to identify significant associations. Additionally, global DNA methylation is not a very sensitive method for identifying or reflecting real biological changes, which might be one of the reasons findings from the included global methylation studies were inconsistent. In addition, future research should note combined exposure to different air pollutants, as indicated by the finding that joint exposure (PM_2.5_, PM_10_, SO_2_, NO_2_, and CO) could induce multiple DMRs.

We found that hypermethylation of *SOX2* was influenced by ambient PM_2.5_ exposure in candidate-gene studies, although this was based on only two observational studies. The transcription factor *SOX2* is essential for embryonic development, and plays a crucial role in controlling several features of cancer cells, such as proliferation, migration, invasion and metastasis, thus resulting in cancer pathogenesis [79]. *SOX2* may present a potential molecular marker for lung cancer [80]. Methylation of the *SOX2* promoter is thought to be useful for early prediction of lung cancer risk in non-smokers exposed to air pollution. Moreover, in oesophageal squamous cell carcinomas, *SOX2* overexpression with *p53* and *p16* inactivation promotes chromatin remodelling by shaping the epigenome [81]. Yachida and colleagues also found that the *SOX2* gene was overexpressed in neuroendocrine carcinomas of the gastrointestinal system due to hypermethylation of the promoter region [82]. These findings indicate that hypermethylation of *SOX2* could serve as a biomarker for diseases (*e.g.* lung cancers and gastrointestinal diseases) and as a new avenue for exploring the mechanisms underlying the impact of environmental exposures on cancers. We also showed that total suspended particles were linked to epigenetic changes in tumour suppressor genes in breast tumours, such as *SCGB3A1*, *SYK*, and *CCND2*, suggesting that assessing methylation of these sites is warranted for cancer therapy.

EWAS is a commonly used strategy for discovering DNA methylation variations across conditions and phenotypes [83]. Methylation in *PES1* was reported to be associated with both ambient PM_2.5_ and PM_10_ exposure [11]. The *PES1* encodes a nuclear protein that contains the breast cancer-associated gene 1 C-terminal interaction domain, which plays a key role in the proliferation and carcinogenicity of breast cancer. *PES1* is overexpressed in original breast tumours compared with normal mammary tissues, and knockdown of *PES1* inhibits the growth of breast cancer cells [84]. We also identified that wildfire-related PM_2.5_-associated CpGs (mapped to genes) are enriched for pathways regarding inflammatory regulation and platelet activity [61]. Methylation of cg00475490 (*PRSS23*) by PCBs and cg08500171 (*BAT2*) by NO_2_ were successfully replicated in external validation cohorts. Originally discovered as an ovarian protease [85], *PRSS23* has been demonstrated to be up-regulated by oestrogen receptor alpha and to stimulate the proliferation of breast cancer cells [86]. *BAT2* encodes a mitochondrial protein that is expressed in all organs except liver cells and is essential for the branched-chain amino acid catabolic pathway [87]. Studies have shown that missense variants in *BAT2* have been linked to lung cancer in multiple populations [88,89]. However, the identified CpGs/DMRs were heterogeneous across the included studies, because these studies are likely to report significant EWAS signals, making it difficult to identify overlapping EWAS findings across different studies. In addition, as noted with the global methylation studies, differences in the data sources and chemical composition of PM and NOx in different populations will contribute to the relative diversity of observations relating to PM and NOx, as suggested by the lack of common EWAS signals of the top PM_2.5_- and NO_2_-associated CpGs [33].

PM exposure can induce post-translational histone modifications, including H3K9me2, H3K4me3, H3K27me3, H3K79me3, and H3K4me2 [90]. While prenatal exposures were outside the scope of our search, it has been reported that air pollution-induced alterations in cord plasma histone H3 modifications during early life might be an indicator that circulating histones could be a risk factor in the development of air pollution-related diseases (*e.g.* lung cancer and stroke) in later life [91,92]. We found that ambient PM_2.5_ exposure in adults was inversely associated with histone modification (mainly H3K9me2). It has been shown that reactive oxygen species generated by PM_2.5_ alter H3K27 acetylation levels, leading to a global increase in enhancer-associated H3K27ac and further activating CXC-chemokine receptors, which may be related to changes in lung function through inflammatory pathways [93]. It should be noted that there are quite limited studies to elucidate the evidence between ambient air pollution and histone modifications. Given that DNA methylation and histone modification are key epigenetic alterations in cellular functions, prospective studies with larger sample sizes are warranted to assess the long-term consequences of these pathways.

We also identified occupational exposure studies related to pollutants in ambient air. Considering that the health impacts of exposures in ambient and occupational settings can differ in nature, including concentration, duration of exposure, composition, and co-exposure to other pollutants and stressors, we reported those occupational findings separately. The overall evidence from the identified studies suggests that long-term occupational exposure to inhaled pollutants could also affect epigenetic changes in a variety of genes (*APC*, *p16*, *p53*, *RASSF1A*, *NOS3*, *EDN1*, *F2RL3*, *AHRR*, H3K4me2, and H3K9ac). Aberrant DNA methylation of the *APC* could act as a strong predictor for cancer progression via the regulation of programmed cell death [94]. *NOS3* encodes endothelial nitric oxide synthase, and nitric oxide plays an important and diverse signalling role in the cardiovascular system, contributing to the regulation of vascular tone. Evidence has reported that higher PM_2.5_ exposure was associated with DNA methylation of several CpG loci in the *NOS3* gene, which indicates that these contaminants may change nitric oxide production through an epigenetic mechanism [95]. Accumulating studies consistently showed that methylation of *F2RL3* and *AHRR* were the top-ranked signals associated with tobacco smoking, and the hypomethylation changes have been linked to increased risk of lung cancer and myocardial infarction [96–98]. One of the most common occupational exposures identified was PM exposure among steel workers, which did not cause histone alterations in H3K4me2 and H3K9ac. However, PM is rich in metals, which may instigate free radical generation, promoting pathophysiological changes driven by oxidative stress and inflammation [99]. Mechanistic studies together with new cohort studies exploring long-term health outcomes are required to further address these findings.

Current studies conducted on air pollution-induced epigenetic alterations have several challenges and limitations. In the context of air pollution, there will inevitably be some degree of measurement and attribution error in assigning individual air pollution concentrations. In line with this, among the 23 studies at overall high risk of bias assessed by the ROBINS-E tool, most of which are predominantly attributed to the high risk of measurement of the exposure bias. Land-use regression modelling based on actual measurements, combined with predictor variables such as nearby population/household density, can address this limitation to some extent [100,101]. In addition, people are exposed to mixtures of air pollution (such as differences in PM sources and sizes, and exposure to co-pollutants) [102], and it is difficult to separate the effects of closely correlated air pollutants, for example, PM and NO_2_ derived from traffic. Some studies assessed pollutant exposure by measuring the pollutant or a metabolite in a biological sample, such as blood or urine. There will be inherent differences in the biokinetics of the pollutant between individuals, but more so, the origin of the pollutant (*i.e.* PCBs) may not be certain and could have arisen from ingestion of the pollutant in water or food rather than inhalation. Regarding the measurement of epigenetic modifications, the accuracy of laboratory measurements, unmeasured batch effects, and the testing regions across different methylation arrays can vary widely, leading to inconsistencies in findings. In addition, four studies used tissue (brain and breast) and semen samples to measure epigenetic changes, indicating blood-specific (*SOX2, DLEC1,* and *AHRR*), breast tissue-specific (*SCGB3A1, SYK, and CCND2*), and sperm-specific (*ACHE*) gene methylation patterns related to different ambient air pollution exposures. Also, an inconsistency in histone modification of the gene H3K4me2 was observed in those participants exposed to PM, in both blood and brain tissue samples. Furthermore, a small number of observational studies were identified. In addition, in most studies, only significant EWAS signals or candidate genes were reported, which limited our ability to perform quantitative stratified meta-analysis and sensitivity analyses on overlapping CpG sites or genes associated with the same air pollutant. Most of the identified observational studies have relatively small sample sizes (range = 23–8397 participants), which likely contributed to the frequently observed trends that did not reach statistical significance. Lastly, most studies failed to control for season and temperature as confounders, and eight studies did not adjust for smoking status. DNA methylation at specific sites is robustly associated with environmental exposures, such as tobacco smoking and obesity [103]. Therefore, these should be considered as important sources of confounding in future studies.

The strengths of our review include its extensive coverage and its in-depth, reproducible evaluation of the existing evidence. Our synthesis of current evidence suggested certain patterns of epigenetic modifications altered by pollutants among public and occupation-based populations. Importantly, we highlighted locus-specific epigenome alterations that have been successfully validated in external data sets. These changes are significant, directing further studies to important mechanistic pathways and to those susceptible to the adverse effects of air pollution. We systematically assessed all included studies for risk of bias using the ROBINS-E tool, particularly for the evaluation of air pollution measurement, epigenetic alterations, and control of confounding factors. Meanwhile, several limitations of our review should also be considered. First, the search strategy was not designed to capture all occupational exposures. A purpose-designed review examining ambient air pollution and other occupational exposures may help identify patterns of epigenetic challenges associated with distinct characteristics of the pollutants in these settings, and potentially provide a mechanistic bridge between those exposures and diseases common to those occupations. Second, we only assessed participants aged <65 years to minimise bias due to ageing-associated epigenetic changes. Future research in the elderly is needed, as they are more vulnerable to air pollution exposure and may exhibit different patterns of epigenetic alterations [104]. Finally, we could not perform meta-analyses due to the highly heterogeneous study methodologies and the use of different epigenetic endpoints.

## CONCLUSIONS

We found that exposures to ambient air pollution, notably PM, were associated with locus-specific DNA methylation changes in adults. However, these changes need to be balanced against many studies that did not consistently cause epigenetic alterations. Although the number of studies was small for specific air-pollutant-epigenetic pairs, the observed epigenetic changes can be linked to epigenetic changes in genes regulating pathways related to cancers. Different patterns of gene-specific DNA methylation were induced by ambient air pollution and occupational exposure, while commonalities emerged across broad classes of air pollutants in both ambient and occupational studies. Future studies with larger sample sizes are needed to identify the robustness of air pollution induced blood- and tissue-specific CpG sites or regions among different populations and assess the potential biological response of epigenetic alterations between air pollution and specific health outcomes, beyond cancers and cardiorespiratory traits, considering the growing links between air pollution and diseases in other organ systems.

## Additional material


Online Supplementary Document

## References

[1] Global Burden of Disease 2021 Forecasting Collaborators Burden of disease scenarios for 204 countries and territories, 2022-2050: a forecasting analysis for the Global Burden of Disease Study 2021. Lancet. 2024;403:2204–56. 10.1016/S0140-6736(24)00685-8 38762325 PMC11121021

[2] World Health Organization. Ambient (outdoor) air pollution. 2024. Available: https://www.who.int/news-room/fact-sheets/detail/ambient-(outdoor)-air-quality-and-health. Accessed: 12 December 2025.

[3] Schraufnagel DE Balmes JR Cowl CT De Matteis S Jung SH Mortimer K Air Pollution and Noncommunicable Diseases: A Review by the Forum of International Respiratory Societies’ Environmental Committee, Part 2: Air Pollution and Organ Systems. Chest. 2019;155:417–26. 10.1016/j.chest.2018.10.041 30419237 PMC6904854

[4] Gref A Merid SK Gruzieva O Ballereau S Becker A Bellander T Genome-Wide Interaction Analysis of Air Pollution Exposure and Childhood Asthma with Functional Follow-up. Am J Respir Crit Care Med. 2017;195:1373–83. 10.1164/rccm.201605-1026OC 27901618 PMC5443897

[5] Lee HT Oh S Ro DH Yoo H Kwon YW The Key Role of DNA Methylation and Histone Acetylation in Epigenetics of Atherosclerosis. J Lipid Atheroscler. 2020;9:419–34. 10.12997/jla.2020.9.3.419 33024734 PMC7521974

[6] de F C Lichtenfels AJ van der Plaat DA de Jong K van Diemen CC Postma DS Nedeljkovic I Long-term Air Pollution Exposure, Genome-wide DNA Methylation and Lung Function in the LifeLines Cohort Study. Environ Health Perspect. 2018;126:027004. 10.1289/EHP2045 29410382 PMC6047358

[7] Mattei AL Bailly N Meissner A DNA methylation: a historical perspective. Trends Genet. 2022;38:676–707. 10.1016/j.tig.2022.03.010 35504755

[8] Baccarelli A Bollati V Epigenetics and environmental chemicals. Curr Opin Pediatr. 2009;21:243–51. 10.1097/MOP.0b013e32832925cc 19663042 PMC3035853

[9] Angelopoulou E Paudel YN Papageorgiou SG Piperi C Environmental Impact on the Epigenetic Mechanisms Underlying Parkinson’s Disease Pathogenesis: A Narrative Review. Brain Sci. 2022;12:175. 10.3390/brainsci12020175 35203939 PMC8870303

[10] Nwanaji-Enwerem JC Dai L Colicino E Oulhote Y Di Q Kloog I Associations between long-term exposure to PM(2.5) component species and blood DNA methylation age in the elderly: The VA normative aging study. Environ Int. 2017;102:57–65. 10.1016/j.envint.2016.12.024 28284819 PMC5396466

[11] Plusquin M Guida F Polidoro S Vermeulen R Raaschou-Nielsen O Campanella G DNA methylation and exposure to ambient air pollution in two prospective cohorts. Environ Int. 2017;108:127–36. 10.1016/j.envint.2017.08.006 28843141 PMC6139298

[12] Wu Y Qie R Cheng M Zeng Y Huang S Guo C Air pollution and DNA methylation in adults: A systematic review and meta-analysis of observational studies. Environ Pollut. 2021;284:117152. 10.1016/j.envpol.2021.117152 33895575

[13] Miller JL Grant PA The role of DNA methylation and histone modifications in transcriptional regulation in humans. Subcell Biochem. 2013;61:289–317. 10.1007/978-94-007-4525-4_13 23150256 PMC6611551

[14] Hou L Zhang X Wang D Baccarelli A Environmental chemical exposures and human epigenetics. Int J Epidemiol. 2012;41:79–105. 10.1093/ije/dyr154 22253299 PMC3304523

[15] Ke Q Davidson T Chen H Kluz T Costa M Alterations of histone modifications and transgene silencing by nickel chloride. Carcinogenesis. 2006;27:1481–8. 10.1093/carcin/bgl004 16522665

[16] Schnekenburger M Talaska G Puga A Chromium cross-links histone deacetylase 1-DNA methyltransferase 1 complexes to chromatin, inhibiting histone-remodeling marks critical for transcriptional activation. Mol Cell Biol. 2007;27:7089–101. 10.1128/MCB.00838-07 17682057 PMC2168892

[17] Cantone L Nordio F Hou L Apostoli P Bonzini M Tarantini L Inhalable metal-rich air particles and histone H3K4 dimethylation and H3K9 Acetylation in a Cross-sectional Study of Steel Workers. Environ Health Perspect. 2011;119:964–9. 10.1289/ehp.1002955 21385672 PMC3222996

[18] Henikoff S Shilatifard A Histone modification: cause or cog? Trends Genet. 2011;27:389–96. 10.1016/j.tig.2011.06.006 21764166

[19] Arita A Shamy MY Chervona Y Clancy HA Sun H Hall MN The effect of exposure to carcinogenic metals on histone tail modifications and gene expression in human subjects. J Trace Elem Med Biol. 2012;26:174–8. 10.1016/j.jtemb.2012.03.012 22633395 PMC3620044

[20] Howe CG Gamble MV Influence of Arsenic on Global Levels of Histone Posttranslational Modifications: a Review of the Literature and Challenges in the Field. Curr Environ Health Rep. 2016;3:225–37. 10.1007/s40572-016-0104-1 27352015 PMC4967376

[21] Zheng Y Sanchez-Guerra M Zhang Z Joyce BT Zhong J Kresovich JK Traffic-derived particulate matter exposure and histone H3 modification: A repeated measures study. Environ Res. 2017;153:112–9. 10.1016/j.envres.2016.11.015 27918982 PMC5605137

[22] Page MJ McKenzie JE Bossuyt PM Boutron I Hoffmann TC Mulrow CD The PRISMA 2020 statement: an updated guideline for reporting systematic reviews. BMJ. 2021;372. 33782057 10.1136/bmj.n71 PMC8005924

[23] Sterne JA Hernán MA Reeves BC Savović J Berkman ND Viswanathan M ROBINS-I: a tool for assessing risk of bias in non-randomised studies of interventions. BMJ. 2016;355:i4919. 10.1136/bmj.i4919 27733354 PMC5062054

[24] Alegría-Torres JA Barretta F Batres-Esquivel LE Carrizales-Yanez L Perez-Maldonado IN Baccarelli A Epigenetic markers of exposure to polycyclic aromatic hydrocarbons in Mexican brickmakers: a pilot study. Chemosphere. 2013;91:475–80. 10.1016/j.chemosphere.2012.11.077 23305747

[25] Barbosa E Dos Santos ALA Peteffi GP Schneider A Muller D Rovaris D Increase of global DNA methylation patterns in beauty salon workers exposed to low levels of formaldehyde. Environ Sci Pollut Res Int. 2019;26:1304–14. 10.1007/s11356-018-3674-7 30421373

[26] Calderón-Garcidueñas L Herrera-Soto A Jury N Maher BA Gonzalez-Maciel A Reynoso-Robles R Reduced repressive epigenetic marks, increased DNA damage and Alzheimer’s disease hallmarks in the brain of humans and mice exposed to particulate urban air pollution. Environ Res. 2020;183:109226. 10.1016/j.envres.2020.109226 32045727

[27] Callahan CL Bonner MR Nie J Han D Wang Y Tao MH Lifetime exposure to ambient air pollution and methylation of tumor suppressor genes in breast tumors. Environ Res. 2018;161:418–24. 10.1016/j.envres.2017.11.040 29197760 PMC5747980

[28] Cantone L Tobaldini E Favero C Albetti B Sacco RM Torgano G Particulate air pollution, clock gene methylation, and stroke: Effects on stroke severity and disability. Int J Mol Sci. 2020;21:3090. 10.3390/ijms21093090 32349365 PMC7247663

[29] Cheng Y Tang Q Lu Y Li M Zhou Y Wu P Semen quality and sperm DNA methylation in relation to long-term exposure to air pollution in fertile men: A cross-sectional study. Environ Pollut. 2022;300:118994. 10.1016/j.envpol.2022.118994 35167929

[30] Chi GC Liu Y MacDonald JW Barr RG Donohue KM Hensley MD Long-term outdoor air pollution and DNA methylation in circulating monocytes: Results from the Multi-Ethnic Study of Atherosclerosis (MESA). Environ Health. 2016;15:119. 10.1186/s12940-016-0202-4 27903268 PMC5131503

[31] Chi GC Liu Y MacDonald JW Reynolds LM Enquobahrie DA Fitzpatrick AL Epigenome-wide analysis of long-term air pollution exposure and DNA methylation in monocytes: results from the Multi-Ethnic Study of Atherosclerosis. Epigenetics. 2022;17:297–313. 10.1080/15592294.2021.1900028 33818294 PMC8920186

[32] Chou YH Tantoh DM Wu MC Tyan YS Chen PH Nfor ON PM2.5 exposure and DLEC1 promoter methylation in Taiwan Biobank participants. Environ Health Prev Med. 2020;25:68. 10.1186/s12199-020-00909-x 33153431 PMC7646067

[33] Eze IC Jeong A Schaffner E Rezwan FI Ghantous A Foraster M Genome-wide DNA methylation in peripheral blood and long-term exposure to source-specific transportation noise and air pollution: The SAPALDIA study. Environ Health Perspect. 2020;128:67003. 10.1289/EHP6174 32484729 PMC7263738

[34] Fiorito G Vlaanderen J Polidoro S Gulliver J Galassi C Ranzi A Oxidative stress and inflammation mediate the effect of air pollution on cardio- and cerebrovascular disease: A prospective study in nonsmokers. Environ Mol Mutagen. 2018;59:234–46. 10.1002/em.22153 29114965

[35] Ghosh M Oner D Poels K Tabish AM Vlaanderen J Pronk A Changes in DNA methylation induced by multi-walled carbon nanotube exposure in the workplace. Nanotoxicology. 2017;11:1195–210. 10.1080/17435390.2017.1406169 29191063

[36] Gondalia R Baldassari A Holliday KM Justice AE Mendez-Giraldez R Stewart JD Methylome-wide association study provides evidence of particulate matter air pollution-associated DNA methylation. Environ Int. 2019;132:104723. 10.1016/j.envint.2019.03.071 31208937 PMC6754789

[37] Goobie GC Li X Ryerson CJ Carlsten C Johannson KA Fabisiak JP PM2.5 and constituent component impacts on global DNA methylation in patients with idiopathic pulmonary fibrosis. Environ Pollut. 2022;318:120942. 10.1016/j.envpol.2022.120942 36574806

[38] Guo L Wang Y Yang X Wang T Yin J Zhao L Aberrant mitochondrial DNA methylation and declined pulmonary function in a population with polycyclic aromatic hydrocarbon composition in particulate matter. Environ Res. 2022;214:113797. 10.1016/j.envres.2022.113797 35779619

[39] Honkova K Rossnerova A Chvojkova I Milcova A Margaryan H Pastorkova A Genome-Wide DNA Methylation in Policemen Working in Cities Differing by Major Sources of Air Pollution. Int J Mol Sci. 2022;23:1666. 10.3390/ijms23031666 35163587 PMC8915177

[40] Hou L Zhang X Tarantini L Nordio F Bonzini M Angelici L Ambient PM exposure and DNA methylation in tumor suppressor genes: a cross-sectional study. Part Fibre Toxicol. 2011;8:25. 10.1186/1743-8977-8-25 21878113 PMC3180673

[41] Hou L Zhang X Zheng Y Wang S Dou C Guo L Altered methylation in tandem repeat element and elemental component levels in inhalable air particles. Environ Mol Mutagen. 2014;55:256–65. 10.1002/em.21829 24273195 PMC4001244

[42] Hu G Li P Cui X Li Y Zhang J Zhai X Cr(VI)-induced methylation and down-regulation of DNA repair genes and its association with markers of genetic damage in workers and 16HBE cells. Environ Pollut. 2018;238:833–43. 10.1016/j.envpol.2018.03.046 29627753

[43] Issah I Arko-Mensah J Rozek LS Rentschler K Agyekum TP Dwumoh D Association between global DNA methylation (LINE-1) and occupational particulate matter exposure among informal electronic-waste recyclers in Ghana. Int J Environ Health Res. 2022;32:2406–24. 10.1080/09603123.2021.1969007 34404291

[44] Kile ML Fang S Baccarelli AA Tarantini L Cavallari J Christiani DC A panel study of occupational exposure to fine particulate matter and changes in DNA methylation over a single workday and years worked in boilermaker welders. Environ Health. 2013;12:47. 10.1186/1476-069X-12-47 23758843 PMC3700827

[45] Li J Xing X Zhang X Liang B He Z Gao C Enhanced H3K4me3 modifications are involved in the transactivation of DNA damage responsive genes in workers exposed to low-level benzene. Environ Pollut. 2018;234:127–35. 10.1016/j.envpol.2017.11.042 29175474

[46] Liu K Jiang J Lin Y Liu W Zhu X Zhang Y Exposure to polycyclic aromatic hydrocarbons, DNA methylation and heart rate variability among non-current smokers. Environ Pollut. 2021;288:117777. 10.1016/j.envpol.2021.117777 34265559

[47] Messingschlager M Bartel-Steinbach M Mackowiak SD Denkena J Bieg M Klos M Genome-wide DNA methylation sequencing identifies epigenetic perturbations in the upper airways under long-term exposure to moderate levels of ambient air pollution. Environ Res. 2023;233:116413. 10.1016/j.envres.2023.116413 37343754

[48] Mu G Nie X Yang S Ye Z Cheng M Fan L PM2.5-related DNA methylation and the association with lung function in non-smokers. Environ Pollut. 2023;316:120700. 10.1016/j.envpol.2022.120700 36403874

[49] Munnia A Bollati V Russo V Ferrari L Ceppi M Bruzzone M Traffic-Related Air Pollution and Ground-Level Ozone Associated Global DNA Hypomethylation and Bulky DNA Adduct Formation. Int J Mol Sci. 2023;24:2041. 10.3390/ijms24032041 36768368 PMC9916664

[50] Plusquin M Guida F Polidoro S Vermeulen R Raaschou-Nielsen O Campanella G DNA methylation and exposure to ambient air pollution in two prospective cohorts. Environ Int. 2017;108:127–36. 10.1016/j.envint.2017.08.006 28843141 PMC6139298

[51] Sayols-Baixeras S Fernandez-Sanles A Prats-Uribe A Subirana I Plusquin M Kunzli N Association between long-term air pollution exposure and DNA methylation: The REGICOR study. Environ Res. 2019;176:108550. 10.1016/j.envres.2019.108550 31260916

[52] Silva IR Ramos M Arantes L Lengert AVH Oliveira MA Cury FP Evaluation of DNA Methylation Changes and Micronuclei in Workers Exposed to a Construction Environment. Int J Environ Res Public Health. 2019;16:902. 10.3390/ijerph16060902 30871143 PMC6466300

[53] Song X Miao M Zhou X Li D Tian Y Liang H Bisphenol A Exposure and Sperm ACHE Hydroxymethylation in Men. Int J Environ Res Public Health. 2019;16:152. 10.3390/ijerph16010152 30626059 PMC6339044

[54] Tantoh DM Lee KJ Nfor ON Liaw YC Lin C Chu HW Methylation at cg05575921 of a smoking-related gene (AHRR) in non-smoking Taiwanese adults residing in areas with different PM2.5 concentrations. Clin Epigenetics. 2019;11:69. 10.1186/s13148-019-0662-9 31060609 PMC6503351

[55] Tantoh DM Wu MF Ho CC Lung CC Lee KJ Nfor ON SOX2 promoter hypermethylation in non-smoking Taiwanese adults residing in air pollution areas. Clin Epigenetics. 2019;11:46. 10.1186/s13148-019-0647-8 30867047 PMC6416982

[56] Tao MH Zhou J Rialdi AP Martinez R Dabek J Scelo G Indoor air pollution from solid fuels and peripheral Blood DNA methylation: Findings from a population study in Warsaw, Poland. Environ Res. 2014;134:325–30. 10.1016/j.envres.2014.08.017 25199973

[57] Tarantini L Bonzini M Tripodi A Angelici L Nordio F Cantone L Blood hypomethylation of inflammatory genes mediates the effects of metal-rich airborne pollutants on blood coagulation. Occup Environ Med. 2013;70:418–25. 10.1136/oemed-2012-101079 23476046 PMC3963398

[58] Wang C O’Brien KM Xu Z Sandler DP Taylor JA Weinberg CR Long-term ambient fine particulate matter and DNA methylation in inflammation pathways: results from the Sister Study. Epigenetics. 2020;15:524–35. 10.1080/15592294.2019.1699894 31822152 PMC7188394

[59] Wang M Zhao J Wang Y Mao Y Zhao X Huang P Genome-wide DNA methylation analysis reveals significant impact of long-term ambient air pollution exposure on biological functions related to mitochondria and immune response. Environ Pollut. 2020;264:114707. 10.1016/j.envpol.2020.114707 32388307

[60] White AJ Kresovich JK Keller JP Xu Z Kaufman JD Weinberg CR Air pollution, particulate matter composition and methylation-based biologic age. Environ Int. 2019;132:105071. 10.1016/j.envint.2019.105071 31387022 PMC6754788

[61] Xu R Li S Wu Y Yue X Wong EM Southey MC Wildfire-related PM2.5 and DNA methylation: An Australian twin and family study. Environ Int. 2023;171:107704. 10.1016/j.envint.2022.107704 36542997

[62] Xu Y Li H Hedmer M Hossain MB Tinnerberg H Broberg K Occupational exposure to particles and mitochondrial DNA - relevance for blood pressure. Environ Health. 2017;16:22. 10.1186/s12940-017-0234-4 28274239 PMC5343309

[63] Yadav S Longkumer I Garg PR Joshi S Rajkumari S Devi NK Association of air pollution and homocysteine with global DNA methylation: A population-based study from North India. PLoS One. 2021;16:e0260860. 10.1371/journal.pone.0260860 34855899 PMC8638980

[64] Zhang H Li X Ge L Yang J Sun J Niu Q Methylation of CpG island of p14(ARK), p15(INK4b) and p16(INK4a) genes in coke oven workers. Hum Exp Toxicol. 2015;34:191–7. 10.1177/0960327114533576 24837742

[65] Yang J Liu Y Zhang H Zhang H Wang W Fan Y Urinary 1-hydroxypyrene and smoking are determinants of LINE-1 and AhRR promoter methylation in coke oven workers. Mutat Res Genet Toxicol Environ Mutagen. 2018;826:33–40. 10.1016/j.mrgentox.2018.01.001 29412867

[66] Alhamdow A Essig YJ Krais AM Gustavsson P Tinnerberg H Lindh CH Fluorene exposure among PAH-exposed workers is associated with epigenetic markers related to lung cancer. Occup Environ Med. 2020;77:488–95. 10.1136/oemed-2020-106413 32385190 PMC7306866

[67] Curtis SW Cobb DO Kilaru V Terrell ML Marder ME Barr DB Genome-wide DNA methylation differences and polychlorinated biphenyl (PCB) exposure in a US population. Epigenetics. 2021;16:338–52. 10.1080/15592294.2020.1795605 32660331 PMC7901541

[68] Jiménez-Garza O Baccarelli AA Byun HM Márquez-Gamiño S Barrón-Vivanco BS Albores A CYP2E1 epigenetic regulation in chronic, low-level toluene exposure: Relationship with oxidative stress and smoking habit. Toxicol Appl Pharmacol. 2015;286:207–15. 10.1016/j.taap.2015.04.016 25963742

[69] Jiménez-Garza O Guo L Byun HM Carrieri M Bartolucci GB Barrón-Vivanco BS Aberrant promoter methylation in genes related to hematopoietic malignancy in workers exposed to a VOC mixture. Toxicol Appl Pharmacol. 2018;339:65–72. 10.1016/j.taap.2017.12.002 29217486

[70] Pittman GS Wang X Campbell MR Coulter SJ Olson JR Pavuk M Polychlorinated biphenyl exposure and DNA methylation in the Anniston Community Health Survey. Epigenetics. 2020;15:337-57. 10.1080/15592294.2019.1666654 31607210 PMC7153539

[71] Su CL Tantoh DM Chou YH Wang L Ho CC Chen PH Blood-Based SOX2-Promoter Methylation in Relation to Exercise and PM2.5 Exposure among Taiwanese Adults. Cancers (Basel). 2020;12:504. 10.3390/cancers12020504 32098209 PMC7072405

[72] Xing C Chen Q Li G Zhang L Zheng M Zou Z Microsomal epoxide hydrolase (EPHX1) polymorphisms are associated with aberrant promoter methylation of ERCC3 and hematotoxicity in benzene-exposed workers. Environ Mol Mutagen. 2013;54:397–405. 10.1002/em.21786 23797950

[73] Zheng M Lin F Hou F Li G Zhu C Xu P Association between Promoter Methylation of Gene ERCC3 and Benzene Hematotoxicity. Int J Environ Res Public Health. 2017;14:921. 10.3390/ijerph14080921 28813025 PMC5580623

[74] Li Z Su Q Xu R Peng J Wang Z Zhu X Effect of acute PM2.5 exposure on PTGS2 and RNA m6A modification. Environ Pollut. 2023;335:122264. 10.1016/j.envpol.2023.122264 37499968

[75] Portela A Esteller M Epigenetic modifications and human disease. Nat Biotechnol. 2010;28:1057-68. 10.1038/nbt.1685 20944598

[76] Thatcher TH Woeller CF McCarthy CE Sime PJ Quenching the fires: Pro-resolving mediators, air pollution, and smoking. Pharmacol Ther. 2019;197:212–24. 10.1016/j.pharmthera.2019.02.001 30759375 PMC6537608

[77] Baccarelli A Wright RO Bollati V Tarantini L Litonjua AA Suh HH Rapid DNA methylation changes after exposure to traffic particles. Am J Respir Crit Care Med. 2009;179:572–8. 10.1164/rccm.200807-1097OC 19136372 PMC2720123

[78] Chen J Hoek G Long-term exposure to PM and all-cause and cause-specific mortality: A systematic review and meta-analysis. Environ Int. 2020;143:105974. 10.1016/j.envint.2020.105974 32703584

[79] Novak D Hüser L Elton JJ Umansky V Altevogt P Utikal J SOX2 in development and cancer biology. Semin Cancer Biol. 2020;67:74–82. 10.1016/j.semcancer.2019.08.007 31412296

[80] Ying J Shi C Li CS Hu LP Zhang WD Expression and significance of SOX2 in non-small cell lung carcinoma. Oncol Lett. 2016;12:3195–8. 10.3892/ol.2016.5065 27899982 PMC5103921

[81] Wu Z Zhou J Zhang X Zhang Z Xie Y Liu JB Reprogramming of the esophageal squamous carcinoma epigenome by SOX2 promotes ADAR1 dependence. Nat Genet. 2021;53:881–94. 10.1038/s41588-021-00859-2 33972779 PMC9124436

[82] Yachida S Totoki Y Noë M Nakatani Y Horie M Kawasaki K Comprehensive Genomic Profiling of Neuroendocrine Carcinomas of the Gastrointestinal System. Cancer Discov. 2022;12:692–711. 10.1158/2159-8290.CD-21-0669 34880079 PMC9394397

[83] Xiong Z Yang F Li M Ma Y Zhao W Wang G EWAS Open Platform: integrated data, knowledge and toolkit for epigenome-wide association study. Nucleic Acids Res. 2022;50:D1004–9. 10.1093/nar/gkab972 34718752 PMC8728289

[84] Li J Yu L Zhang H Wu J Yuan J Li X Down-regulation of pescadillo inhibits proliferation and tumorigenicity of breast cancer cells. Cancer Sci. 2009;100:2255–60. 10.1111/j.1349-7006.2009.01325.x 19764998 PMC11159139

[85] Miyakoshi K Murphy MJ Yeoman RR Mitra S Dubay CJ Hennebold JD The identification of novel ovarian proteases through the use of genomic and bioinformatic methodologies. Biol Reprod. 2006;75:823–35. 10.1095/biolreprod.106.052290 16870946

[86] Chan HS Chang SJ Wang TY Ko HJ Lin YC Lin KT Serine protease PRSS23 is upregulated by estrogen receptor α and associated with proliferation of breast cancer cells. PLoS One. 2012;7:e30397. 10.1371/journal.pone.0030397 22291950 PMC3264607

[87] Zhang L Zhang Y Hu Z The Effects of Catabolism Relationships of Leucine and Isoleucine with BAT2 Gene of Saccharomyces cerevisiae on High Alcohols and Esters. Genes (Basel). 2022;13:1178. 10.3390/genes13071178 35885961 PMC9321263

[88] Walsh KM Gorlov IP Hansen HM Wu X Spitz MR Zhang H Fine-mapping of the 5p15.33, 6p22.1-p21.31, and 15q25.1 regions identifies functional and histology-specific lung cancer susceptibility loci in African-Americans. Cancer Epidemiol Biomarkers Prev. 2013;22:251–60. 10.1158/1055-9965.EPI-12-1007-T 23221128 PMC3565099

[89] Jin G Zhu M Yin R Shen W Liu J Sun J Low-frequency coding variants at 6p21.33 and 20q11.21 are associated with lung cancer risk in Chinese populations. Am J Hum Genet. 2015;96:832–40. 10.1016/j.ajhg.2015.03.009 25937444 PMC4570553

[90] Kresovich JK Zhang Z Fang F Zheng Y Sanchez-Guerra M Joyce BT Histone 3 modifications and blood pressure in the Beijing Truck Driver Air Pollution Study. Biomarkers. 2017;22:584–93. 10.1080/1354750X.2017.1347961 28678539 PMC5599708

[91] Vrijens K Trippas AJ Lefebvre W Vanpoucke C Penders J Janssen BG Association of Prenatal Exposure to Ambient Air Pollution With Circulating Histone Levels in Maternal Cord Blood. JAMA Netw Open. 2020;3:e205156. 10.1001/jamanetworkopen.2020.5156 32421184 PMC7235690

[92] Abrams ST Zhang N Manson J Liu T Dart C Baluwa F Circulating histones are mediators of trauma-associated lung injury. Am J Respir Crit Care Med. 2013;187:160–9. 10.1164/rccm.201206-1037OC 23220920 PMC3570656

[93] Xu Y Barregard L Nielsen J Gudmundsson A Wierzbicka A Axmon A Effects of diesel exposure on lung function and inflammation biomarkers from airway and peripheral blood of healthy volunteers in a chamber study. Part Fibre Toxicol. 2013;10:60. 10.1186/1743-8977-10-60 24321138 PMC4029460

[94] Goss KH Groden J Biology of the adenomatous polyposis coli tumor suppressor. J Clin Oncol. 2000;18:1967–79. 10.1200/JCO.2000.18.9.1967 10784639

[95] Breton CV Salam MT Wang X Byun HM Siegmund KD Gilliland FD Particulate matter, DNA methylation in nitric oxide synthase, and childhood respiratory disease. Environ Health Perspect. 2012;120:1320–6. 10.1289/ehp.1104439 22591701 PMC3440108

[96] Corbin LJ White SJ Taylor AE Williams CM Taylor K van den Bosch MT Epigenetic Regulation of F2RL3 Associates With Myocardial Infarction and Platelet Function. Circ Res. 2022;130:384–400. 10.1161/CIRCRESAHA.121.318836 35012325 PMC8812435

[97] Zhang Y Yang R Burwinkel B Breitling LP Holleczek B Schöttker B F2RL3 methylation in blood DNA is a strong predictor of mortality. Int J Epidemiol. 2014;43:1215–25. 10.1093/ije/dyu006 24510982 PMC4258765

[98] Lee DH Hwang SH Lim MK Oh JK Song DY Yun EH Performance of urine cotinine and hypomethylation of AHRR and F2RL3 as biomarkers for smoking exposure in a population-based cohort. PLoS One. 2017;12:e0176783. 10.1371/journal.pone.0176783 28453567 PMC5409156

[99] Mercorio R Bonzini M Angelici L Iodice S Delbue S Mariani J Effects of metal-rich particulate matter exposure on exogenous and endogenous viral sequence methylation in healthy steel-workers. Environ Res. 2017;159:452–7. 10.1016/j.envres.2017.08.042 28858759

[100] de Hoogh K Wang M Adam M Badaloni C Beelen R Birk M Development of land use regression models for particle composition in twenty study areas in Europe. Environ Sci Technol. 2013;47:5778–86. 10.1021/es400156t 23651082

[101] Eeftens M Beelen R de Hoogh K Bellander T Cesaroni G Cirach M Development of Land Use Regression models for PM(2.5), PM(2.5) absorbance, PM(10) and PM(coarse) in 20 European study areas; results of the ESCAPE project. Environ Sci Technol. 2012;46:11195–205. 10.1021/es301948k 22963366

[102] Wei Y Qiu X Yazdi MD Shtein A Shi L Yang J The Impact of Exposure Measurement Error on the Estimated Concentration-Response Relationship between Long-Term Exposure to PM2.5 and Mortality. Environ Health Perspect. 2022;130:77006. 10.1289/EHP10389 35904519 PMC9337229

[103] Hannon E Knox O Sugden K Burrage J Wong CCY Belsky DW Characterizing genetic and environmental influences on variable DNA methylation using monozygotic and dizygotic twins. PLoS Genet. 2018;14:e1007544. 10.1371/journal.pgen.1007544 30091980 PMC6084815

[104] Wang K Liu H Hu Q Wang L Liu J Zheng Z Epigenetic regulation of aging: implications for interventions of aging and diseases. Signal Transduct Target Ther. 2022;7:374. 10.1038/s41392-022-01211-8 36336680 PMC9637765

