# Supplementary material for: A systematic review of observational studies on long-term air pollution exposure and epigenetic alterations in adults
Source: J Glob Health. 2026 Mar 20;16:04087. doi: 10.7189/jogh.16.04087 (PMC13002177; doi:10.7189/jogh.16.04087)
Supplement: Online Supplementary Document [file jogh-16-04087-s001.zip › jogh-16-04087-s001.pdf]

Supplement to: Yu L, Zhao Y, Chen W, Yu G, Miller M, Li X, Theodoratou E. A systematic review of observational studies on long-term air pollution exposure and epigenetic alterations in adults. J Glob Health. 2026;16:04087.

Supplementary Tables and Figures

Table S1. Systematic literature review search terms and strategy.

Table S2. Quality assessment criteria for ROBINS-E tool.

Table S3. Study population, exposure characteristics and main findings of 20 occupational exposure studies using global methylation and candidate-gene methylation approaches.

Table S4. Study population, exposure characteristics and main findings of three studies using a histone modification approach.

Table S5. Exposure-related characteristics of studies included in the systematic review.

Table S6. Epigenetic alterations assessment techniques used among included studies.

Table S7. Quality assessment-ROBINS-E tool.

Table S8. Genes methylation investigated in candidate-gene studies.

Table S9. Differentially methylated sites (CpGs) identified from included studies.

Table S10. Differentially methylated sites (CpGs) identified in discovery cohort and replicated in independent external cohorts.

Figure S1. Traffic-light plots of the domain-level judgements for each study.

Table S1. Systematic literature review search terms and strategy.

Databases	Search terms used
**Embase (Ovid)**	1. air pollut*.mp. or air pollution/<br>2. volatile organic compound*.mp. or volatile organic compound/<br>3. benzene/ or benzene.mp. or BTEX.mp.<br>4. carbon monoxide.mp. or carbon monoxide/<br>5. ozone.mp. or ozone/<br>6. dust/ or dust.mp.<br>7. sulfur dioxide/ or SO_2.mp.<br>8. nitrogen dioxide/ or NO_2.mp. or nitrogen oxide/<br>9. PCB.mp. or polychlorinated biphenyl/<br>10. PAH.mp. or polycyclic aromatic hydrocarbon/ or poly* aromatic hydrocarbon.mp.<br>11. black carbon.mp. or black carbon/<br>12. motor vehicle.mp. or motor vehicle/<br>13. diesel exhaust.mp. or exhaust gas/ or environmental exposure/ or environmental pollution/ or environmental pollutants/ or outdoor exposure.mp. or traffic related.mp. or wood smoke.mp.<br>14. particulate matter.mp. or particulate matter/ or PM_{10}.mp. or "$PM_{2.5}$".mp.<br>15. polychlorinated biphenyl.mp. OR polychlorinated biphenyl/<br>16. 2 or 3 or 4 or 5 or 6 or 7 or 8 or 9 or10 or 11 or 12 or 13 or 14 or 15<br>17. DNA methylation.mp. or DNA methylation/ or DNA methylation assay/ or methylation/ or hypermethylation.mp. or hypomethylation.mp.<br>18. epigenetic*.mp. or epigenetics/ or epigenomic*.mp. or epigenomics/ or epigenome-wide.mp. or CpG.mp. or histo ne modification/<br>19. 17 or 18<br>20. 1 and 16<br>21. (1 or 20) and 19
**Medline (Ovid)**	1. air pollut*.mp. or air pollutants/<br>2. volatile organic compound*.mp. or volatile organic compounds/<br>3. benzene/ or benzene.mp. or BTEX.mp.<br>4. carbon monoxide.mp. or carbon monoxide/<br>5. ozone.mp. or ozone/<br>6. dust/ or dust.mp.<br>7. sulfur dioxide/ or SO_2.mp.<br>8. nitrogen dioxide/ or NO_2.mp. or nitrogen oxide/<br>9. PCB.mp. or polychlorinated biphenyl.mp. or polychlorinated biphenyls/<br>10. PAH.mp. or polycyclic aromatic hydrocarbons/ or poly* aromatic hydrocarbon.mp.<br>11. black carbon.mp. or soot/<br>12. motor vehicle.mp. or motor vehicles/<br>13. diesel exhaust.mp. or vehicle emissions/ or environmental exposure/ or environmental pollution/ or environmental pollutants/ or outdoor exposure.mp. or traffic related.mp. or wood smoke.mp.<br>14. particulate matter.mp. or particulate matter/ or PM_{10}.mp. or "$PM_{2.5}$".mp.<br>15. polychlorinated biphenyls.mp. OR polychlorinated biphenyls/<br>16. 2 or 3 or 4 or 5 or 6 or 7 or 8 or 9 or10 or 11 or 12 or 13 or 14 or 15<br>17. DNA methylation.mp. or DNA methylation/ or methylation.mp. or methylation/ or hypermethylation.mp. or hypomethylation.mp.<br>18. epigenetic*.mp. or epigenomic*.mp. or epigenomics/ or epigenome-wide.mp. or CpG.mp. or histone code/ or histone modification.mp.<br>19. 17 or 18<br>20. 1 and 16<br>21. (1 or 20) and 19
**Web of Science**	(TI=("air pollut*") OR AB=("air pollut*") OR (TI=("air pollut*") OR AB=("air pollut*")) AND (TI=("volatile organic compounds" OR "carbon monoxide" OR "ozone" OR "dust" OR "SO_2" OR "NO" OR "NO_2" OR "sulfur dioxide" OR "nitrogen dioxide" OR "nitrogen oxide*" OR "polycyclic aromatic hydrocarbons"

	OR "PAH" OR "polychlorinated Biphenyls" OR "PCB" OR "black carbon" OR "motor vehicle" OR "diesel exhaust" OR "outdoor exposure" OR "environmental exposure" OR "diesel exhaust" OR "environmental pollut*" OR "traffic related" OR "vehicle emissions" OR "wood smoke" OR "particulat*" OR "$PM_{2.5}$" OR "PM_{10}") OR AB=("volatile organic compounds" OR "carbon monoxide" OR "ozone" OR "dust" OR "SO_2" OR "NO" OR "NO_2" OR "sulfur dioxide" OR "nitrogen dioxide" OR "nitrogen oxide*" OR "polycyclic aromatic hydrocarbons" OR "PAH" OR "polychlorinated Biphenyls" OR "PCB" OR "black carbon" OR "motor vehicle" OR "diesel exhaust" OR "outdoor exposure" OR "environmental exposure" OR "diesel exhaust" OR "environmental pollut*" OR "traffic related" OR "vehicle emissions" OR "wood smoke" OR "particulat*" OR "$PM_{2.5}$" OR "PM_{10}"))) AND (TI=("DNA methylation*" OR "hypermethylation" OR "hypomethylation" OR "epigenetic*" OR "epigenomic*" OR "epigenome-wide" OR "CpG" OR "histone modification") OR AB=("DNA methylation*" OR "hypermethylation" OR "hypomethylation" OR "epigenetic*" OR "epigenomic*" OR "epigenome-wide" OR "CpG" OR "histone modification"))

Table S2. Quality assessment criteria for ROBINS-E tool.

Domains	Criteria
Confounding bias	Studies used analysis approaches that avoid confounding by individual-level factors, including socioeconomic status (SES) (e.g. income, education), age, sex, race/ethnicity, seasons, temperature, and smoking status are considered at low risk of bias;<br>Studies adjusted for some indicator(s) of SES, race/ethnicity, seasons, temperature, and smoking status are either in an identifiably (largely) racially homogeneous population are considered at some risk of bias;<br>Studies did not adjust for socioeconomic status (SES), race/ethnicity, seasons, temperature, and smoking status are deemed at high risk of bias;
Exposure classification bias	Long-term exposure (at least one year of exposure) using models with good performance and/or estimated air pollution exposure by utilising monitoring stations in studies are considered at low risk of bias;<br>Long-term exposure studies but assessed by other exposure models or measurement are considered at some risk of bias;<br>Long-term exposure using a model with poor performance or unclear exposure estimation reported in studies are considered high risk of bias.
Missing data bias	Studies that less than 10% of participants or used regional administrative datasets but it is likely to minimally impact results are considered them at low risk of bias;<br>Studies used methods to account for attrition or reported low attrition rates are considered at some risk (more than 10% but less than 20%);<br>Studies that enrolled cohorts of people and did not mention follow-up are considered at high risk of bias.
Measurement of the outcome bias	Explicit methodologies for the samples collection and techniques controls in epigenetic alteration assessment are considered as low risk of bias;<br>Studies did not mention techniques controls in epigenetic alteration assessment are considered as some risk of bias;<br>Studies are not based on explicit methodologies for the samples collection and did not mention techniques controls in epigenetic alteration assessment are considered as high risk of bias.
Measurement of the exposure bias	Exposure assessment was performed using land use regression or dispersion modelling assigned to residence level are considered at low risk of bias.<br>Exposure assessment was performed using other methods such as proximity to traffic, nearest monitoring station, inverse distance weighting, etc. but evaluated at individual residential address, but this error is likely small, these studies are considered at some risk of bias.<br>Studies relied on self-report for exposure assessment or are on area-level geocoding, or if the condition was not clear are considered at high risk of bias.
Selection of reported results	While separate documents outlining pre-defined analysis plans were not available for any of the studies, all indications suggested that the main analyses used were the primary objective of the study. In addition, studies applying multiple correction (e.g., false discovery rate) for reported *P* value were considered at low risk of bias, otherwise were some risk of bias.
Selection of participants	In all studies follow-up began at or close to the start of the exposure window and selection of participants was not based on characteristics observed after the exposure window. Thus, we considered all studies at low risk of bias from such selection.
Overall	Overall risk of bias for each study was then recorded as the highest risk of bias for any domain.

Note: The quality assessment criteria were deduced from original ROBINS-E tool, especially for domines of "exposure classification bias" and "measurement of the outcome". The domine of "exposure classification bias" and "measurement of the outcome bias" were adapted from "Post-exposure interventions" and "measurement of the outcome bias" in original ROBINS-E tool, respectively. In our present systematic review, we take the air pollution measurement (exposure periods and modelling) as the exposure classification rather than passive/active case ascertainment, as well as take epigenetic alteration assessment (samples collection and techniques controls) as the outcomes rather than disease diagnosis in the original ROBINS-E tool.

Table S3. Study population, exposure characteristics and main findings of 20 occupational exposure studies using global methylation and candidate-gene methylation approaches.

First author, year	Location	Study population	Study design	Number of participants	Age of participants (year)	Type of pollutant	Studied region or loci	Main findings and coefficient estimates
Alegría-Torres, 2013	Mexico	Male brick makers	Cross-sectional	39	42.50 ± 15.84	Polycyclic aromatic hydrocarbons (PAHs)	Global DNA methylation: *Alu* and *LINE-1*	1-OHP urine concentration was negatively associated with DNA hypomethylation of the interleukin 12 (β=-1.57; 95% CI: -2.9, -0.23; per µg/g) and p53 gene promoters (β=-2.7; 95% CI: -5.46, 0.06; per µg/g).
Barbosa, 2019	Brazil	Salon workers	Cross-sectional	49	Group A (FA< 0.01 ppm): 31.25 ± 11.09, Group B (0.03 ppm< FA< 0.06 ppm): 33.87 ± 10.79, Group C (0.08 ppm < FA< 0.24 ppm): 29.35 ± 10.13	Formaldehyde (FA)	Global DNA methylation	Low levels of FA exposure could cause a disturbance in DNA methylation (r_s=0.307), leading to epigenetic changes.
Ghosh, 2017	Netherland	MWCNT-exposed workers and unexposed controls	Cross-sectional	67	Exposed: 35.87 ± 6.90, Control: 34.64 ± 8.57	multi-wall carbon nanotubes (MWCNT)	Global DNA methylation: *LINE-1*	No significant changes in Cytosine-phosphate-Guanine (CpG) site methylation were observed for the LINE-1 elements.
Issah, 2022	Ghana	Exposed: electronic-waste recyclers; Control: non-e-waste workers	Cross-sectional	151	electronic-waste recyclers: 25.4 ± 6.3; non-e-waste workers: 32.5 ± 10.4	$PM_{2.5}$, PM_{10}	Global DNA Methylation: *LINE-1*	$PM_{2.5}$ exposure was significantly associated with increased LINE-1 CpG2 DNA methylation (β=0.003; 95% CI: 0.001, 0.006; per µg/m^3) but not with the average of all 4 CpG sites of LINE-1.
Kile, 2013	US	Male boilermaker	Cohort	38	36.0 ± 12.0	$PM_{2.5}$	Global DNA Methylation: *Alu*, *LINE-1*, *iNOS*	$PM_{2.5}$ exposure was not associated with methylation in Alu (β=0.05; se=0.07;

		construction workers						mg/m^3) and LINE-1 (β= -0.12; se=0.10; mg/m^3).
Munnia, 2023	Italy	Exposed: traffic police officers; Control: unexposed subjects	Cross-sectional	140	35.7 ± 5.3	B(a)P, CO, O_3	Global DNA methylation: *LINE-1*, *ALU*, *IL-6*, *iNOS*	Air pollution exposure was associated with a significant reduction in global DNA hypomethylation (95% CI: -2.65, -0.04; per 1.41 $\mu g/m^3$)
Yang, 2018	China	Exposed: coke oven plant workers; Control: subjects from the department of water treatment	Cross-sectional	479	coke oven plant workers: 40 (21–55); Control: 42 (28–53)	polycyclic aromatic hydrocarbons (PAHs)	Global DNA methylation	A high level of urinary 1-hydroxypyrene was associated with a significantly increased risk of hypomethylation of LINE-1 (OR: 1.80; 95% CI: 1.25, 2.60; per µg/mmol creatinine).
Ghosh, 2017	Netherland	MWCNT-exposed workers and unexposed controls	Cross-sectional	67	Exposed: 35.87 ± 6.90, Control: 34.64 ± 8.57	multi-wall carbon nanotubes (MWCNT)	Gene-based: *DNMT1*, *ATM*, *SKI*, and *HDAC4* promoter	A significant change in methylation for *DNMT1*, *ATM*, *SKI*, and *HDAC4* promoter CpGs in multi-wall carbon nanotubes-exposed workers.
Guo, 2022	China	Participants from coke-oven plants in East China	Cross-sectional	768	36	polycyclic aromatic hydrocarbon (PAH)-enriched $PM_{2.5}$	Gene-based: mitochondrial DNA methylation	The methylation of platelet mitochondrial gene MT-*ATP6* was negatively associated with PM exposure.
Hou, 2011	Italy	Male healthy steel workers	Cross-sectional	63	44 (27-55)	PM_{10}, PM_1	Gene-based: p16, *APC*, *p53*, *RASSF1A*	*APC* methylation level was associated with PM_{10} and PM_1, while *RASSF1A*, *p16*, *p53* methylation were not associated with PM_{10} and PM_1.

Hou, 2014	China	The Beijing Truck Driver Air Pollution Study (BTDAS)	Cross-sectional	120	Office workers: 30.3 ± 8.0; Truck drivers: 33.5 ± 5.7	$PM_{2.5}$	Gene-based: *SATα*, *NBL2*, *D4Z4*	*NBL2* methylation was positively associated with concentrations of Si and Ca in truck drivers, and *SATα* methylation was positively associated with concentrations of Si in office workers.
Hu, 2018	China	Exposed: blue-collar workers; Control: worked in administrative offices	Cross-sectional	117	Exposed: 36.0 ± 7.5; Control: 36.0 ± 9.3	Cr (VI)	Gene-based: *MGMT*, *HOGG1*, *XRCC1*, *ERCC3* and *RAD51*	Blood Cr concentration was positively correlated with the methylation level of CpG sites in DNA repair genes among blue-collar workers.
Xu, 2017	Sweden	Male welders	Cross-sectional	228	Welder, median (5-95 percentile): 41 (23 – 60); Control: median (5-95 percentile): 43 (23-56)	Respirable dust	Gene based: MT-TF	Occupational respirable dust was significantly associated with MT-TF methylation.
Zhang, 2015	China	Coke oven workers	Cross-sectional	121	Exposed: 37.05 + 5.65; Control: 38.42 + 3.83	B[a]P	Gene based: p14(*ARK*), p15(*INK4b*) and p16(*INK4a*)	Urinary 1-OH-Py's level and DNA olive tail moment in comet assay were significantly increased in the coke oven workers, and p14(*ARK*), p15(*INK4b*) and p16(*INK4a*) gene methylation rates were also significantly increased.
Alhamdow, 2020	Sweden	Men working in Sweden	Cross-sectional	322	Chimney sweeps: 43 (19-66); Creosote-exposed workers: 32 (22-58); Controls: 43 (20-63)	PAH	Gene based: *AHRR*, *F2RL3*	Chimney sweeps showed the hypomethylation of *F2RL3* (β=-2.7; 95% CI: -3.9, -1.5; per µg/g creatinine) and *AHRR* (β=-7.1; 95% CI: -9.6, -4.7; µg/g creatinine) methylation.

Jiménez-Garza, 2015	Mexico	Exposed: tanneries workers; non-exposed: administrative personnel of universities	Cross-sectional	48	Exposed: 36.7 ± 13.8 non-exposed: 35.9 ± 12.9	Benzene, Ethylbenzene, Toluene	Gene based: *COX-2*, *CYP2E1*, *GSTP1*, *HMOX-1*, *IL-6*, *iNOS*, *SOD1*, *TNFa*, *TOP2A*	A number of CpG sites in candidate-gene showed statistical significance or borderline significance with exposure variables in exposed group.
Jiménez-Garza, 2018	Mexico	Exposed: workers from a leather shoe factory (LS) and a gas station (GS), Control: workers from a public university	Cohort	142	GS: 36.6 ± 11.4, LS: 28.6 ± 10.3, C: 33.3 ± 11.2	Benzene, Toluene, Ethylbenzene	Gene based: *SOD1*, *TOP2A,* and *TNF-α*	Ethylbenzene and toluene associated with *TOP2A* promoter methylation level. Benzene associated with *SOD1* promoter methylation level.
Silva, 2019	Brazil	Barretos Cancer Hospital	Cross-sectional	108	Workers in the exposed construction: 39; nonexposed control administrative group: 32	$PM_{2.5}$ and polycyclic aromatic hydrocarbons (PAHs)	Gene-based: *CDKN2A*, *MLH1*, *APC*	Exposed workers exhibited significantly higher average levels of promoter methylation of *CDKN2A*, *APC*, and *MLH1* genes and increased hypomethylation of the *LINE-1* in comparison to unexposed workers
Tarantini, 2013	Italy	Steel workers	Cross-sectional	63	27-55	PM_{10}, PM_1	Gene based: *NOS3*, *EDN1*	*NOS3* methylation was negatively associated with PM_{10} and PM_1, Zinc exposure was negatively associated with *EDN1* methylation.
Xing, 2013	China	Exposed: benzene-exposed workers; Unexposed:	Cross-sectional	102	Exposed: 56 ± 11; Unexposed: 54 ± 11	Benzene	Gene based: *BLM*, *CYP1A1*, *EPHX1*, *ERCC3*, *NQO1*, *NUDT1*, *p15*, *p16,*	*ERCC3* showed a significant increase in level of methylation in the exposed group. The methylation levels of the other 10 genes were not significantly different across benzene exposure status.

		age-, sex matched general population					*RAD51*, *TP53*, *WRAP53*	
Zheng, 2017	China	Exposed: workers; Control: age- and sex-matched population	Cohort	100	Exposed: 56 ± 11; control: 54 ± 11	Benzene	Gene based: *ERCC3*	Two of analysed ten CpG units in *ERCC3* promoter were significantly increased in the exposed group.

Table S4. Study population, exposure characteristics and main findings of three studies using a histone modification approach.

First author, year	Location	Study population	Study design	Number of participants	Age of participants (year)	Type of pollutant	Studied region or loci	Main findings and coefficient estimates
**Ambient air pollution exposure**								
Calderón-Garcidueñas, 2020	Mexico	Exposed: high pollution-exposure area in Metropolitan Mexico City, Control: low-pollution areas in Mexico	Cohort	23	Exposed: 29.8, Control: 29.3	$PM_{2.5}$	Histone post-translational modifications: H3K9me2 and H3K9me3	A significant decrease in the global intensity of H3K9me2 staining in nuclei of brain samples from MMC urbanites compared to controls.
**Occupational air pollution exposure**								
Cantone, 2011	Italy	Male healthy steel workers	Cross-sectional	63	44	Metal components of PM	Histone modifications: H3K9me2 and H3K9me3	Cumulative exposures to nickel and arsenic were positively correlated with both H3K4me2 (nickel: β=0.16; 95% CI: 0.01, 0.3; arsenic: β=0.16; 95% CI: 0.03, 0.29) H3K9ac (nickel: β=0.27; 95% CI: 0.01, 0.54; arsenic: β=0.28; 95% CI: 0.04, 0.51).
Li, 2018	China	Exposed: low-level benzene exposed workers; Control: control workers	Cross-sectional	269	Benzene-exposed workers :45.95 ± 3.84; Control workers: 46.38 ± 7.10	Benzene	Histone modifications: H3K4me3, H3K36me3, H3K79me2	Global trimethylated histone 3 lysine 4 (H3K4me3) modification was enhanced in the benzene-exposed group and was positively associated with the concentration of urinary SPMA (β=0.103).

Table S5 Exposure-related characteristics of studies included in the systematic review.

First author, year	Exposure definition	Concentration of pollutants	Exposure length	Sample sites	Measure of association	Adjusted confounding factors
**Ambient air pollution exposure**						
Cheng, 2022	Individual 24 state-owned air concentration was identified by the monitoring data	PM_{10}, μg/m^3: 109.69 ± 15.89 (IQR=24.16)	1 year	Semen samples	Multiple linear regression	Age, ethnicity, education, BMI, family income, smoking status, drinking status, *seasons of sample collection and temperature*
Chi, 2016	Monitored by the U.S. Environmental Protection Agency-operated Air Quality System monitors	$PM_{2.5}$, μg/m^3: 10.7 (IQR=2.2), NOx, ppb: 28.7 (IQR=31.9)	1 year	Blood samples	Linear regression models	Age, race/ethnicity, sex, study site, income, education, neighbourhood socioeconomic status factor score, cigarette smoking, second-hand smoke, body mass index, physical activity, methyl nutrient intake, *residual cell contamination by nonmonocytes*, recent infection, and *methylation chip position*
Goobie, 2023	Measured from the Atmospheric Composition Analysis Group online data repository	Simmons Cohort, median (IQR), μg/m^3: 11.2 (9.5-13.4); PFF Cohort, median (IQR): 69 (64-74)	> 1 year	Peripheral blood samples	Multivariable regression models	Age at diagnosis, sex, smoking history, race, baseline FVC, baseline D_LCO, and site in PFF cohort only

Plusquin, 2017	Used models to assess exposure at the baseline address of each cohort member	NOx, $\mu g/m^3$ (5th-95th centile): EPIC-Italy: 92.83 (34.46 - 131.00) EPIC-Netherlands: 29.69 (24.25 - 42.58); NO_2, $\mu g/m^3$: EPIC-Italy: 50.00 (22.26 - 67.92) EPIC-Netherlands: 19.98 (16.74 - 28.91); PM_{10}, $\mu g/m^3$: EPIC-Italy: 46.91 (38.24 - 53.23) EPIC-Netherlands: 24.49 (23.96 - 26.16); $PM_{2.5}$, $\mu g/m^3$: EPIC-Italy: 30.95 (26.74 - 32.73) EPIC-Netherlands: 16.63 (16.40 - 17.12)	2 years and 7 months (Oct 2008 to April 2011)	Peripheral blood leukocytes	Linear regression model	Age, sex, smoking status, and *residual technical confounding* (position of the sample on the micro-array)
Tao, 2014	Calculated the total duration of being exposed to solid fuels as the sum of the differences between the ages at starting and leaving each residence in which solid fuels were used as principle means for cooking or heating	-	27 years	Peripheral blood samples	Linear regression models	Age, education level, BMI, alcohol drinking, dietary folate intake, and exposure to environmental tobacco smoke at workplace or from partner
Wang, 2020	Applied a regionalized national universal kriging model using partial least squares to estimate annual $PM_{2.5}$ concentrations.	$PM_{2.5}$, $\mu g/m^3$: Sub-cohort1: 9.336 ± 2.09； Sub-cohort2: 9.258 ± 2.03	1 year	Whole blood samples	Classical least-squares and quantile regression models	Age at baseline, BMI, alcohol consumption, education, mother’s smoking status, and *cell-type proportions*

Wang, 2020	Derived from environmental monitoring organizations (Jincheng: China National Environmental Monitoring Centre	-	3 years	Whole blood samples	Linear regression models	*Six blood cell subtypes* (B cells, $CD4^+$ T cells, $CD8^+$ T cells, natural killer cells, monocytes, and granulocytes), age, body mass index (BMI), and smoking status
Xu, 2023	Estimated annual average $PM_{2.5}$ for all land grid cells across the globe by combining data from satellite observations, chemical transport models, and ground-based observations	$PM_{2.5}$, μg/m^3: 5.3 ± 2.2	3 years	Whole blood samples	Generalized estimating equation (GEE) model	Age, educational level, marital status, smoking behaviour, alcohol use, area-level socioeconomic status, survey year, survey season, annual average ambient temperature, and annual average relative humidity, and *cell-type proportions*
Yadav, 2021	Distance of residential area from the main road >1km, were categorised into low polluted areas; the villages located on/in proximity to NH-2 (within 1km radius), having more than two pucca roads in each village, factories/brick quarries (within 2km radius), vehicular count >10,000 for 24 hrs, were categorized into high polluted areas	-	2 years	Blood samples	Multiple linear regression	Age, sex, smoking, alcohol consumption, education, occupation, homocysteine, folate, vitamin B12

Callahan, 2018	Obtained from New York State Department of Environmental Conservation air monitors	TSP, µg/m^3: Median = 134	10-20 years	Breast tissue sample	Unconditional logistic regression	Age, second-hand smoke exposure before age 20, current smoking status, and estrogen receptor status
Cantone, 2020	Monitored by the Regional Environmental Protection Agency	-	1 year	Whole blood sample	Linear mixed-effect regression models	Age, sex, smoking habits, and warm/cold months, *run, position and plate*
Chi, 2022	Monitored by the U.S. Environmental Protection Agency-operated Air Quality System monitors	$PM_{2.5}$, µg/m^3: 10.7 (IQR=2.2), NOx, ppb: 28.7 (IQR=31.9)	1 year	Blood samples	Linear regression models	Age, sex, race/ethnicity, household income, education, neighbourhood socioeconomic status, smoking (smoking status and pack years), second-hand smoke, body mass index, recent infection, methyl nutrient intake, physical activity, study site, and *chip position*

Chou, 2020	Estimated participants' exposure from Air Quality Monitoring Database (AQMD)	$PM_{2.5}$, µg/m^3: No regular exercise: 31.551 ± 0.455, Regular exercise: 32.756 ± 0.550<br>SO_2, ppb: No regular exercise: 4.137 ± 0.092, Regular exercise: 4.092 ± 0.099<br>CO, ppm: No regular exercise: 0.560 ± 0.013, Regular exercise: 0.546 ± 0.013<br>O_3, ppb: No regular exercise: 27.860 ± 0.192, Regular exercise: 27.989 ± 0.221<br>NOx, ppb: No regular exercise: 26.411 ± 0.746, Regular exercise: 25.581 ± 0.794	6 years	Blood samples	Multivariate linear regression	Exercise, SO_2, CO, O3, NOx, sex, age, cigarette smoking, BMI, second-hand smoke exposure, and alcohol/betel nut intake, and *cell-type composition*
Song, 2019	Measured urinary BPA concentration of the workers as the BPA exposure	BPA, µg/g Cr: occupational BPA exposure group: 199.13; control: 0.77	average 4 years	Semen samples	Linear regression analysis	Age, history of disease, smoking, and alcohol consumption
Tantoh, 2019	Average $PM_{2.5}$ levels (2006–2011) of the monitors of each area (northern, north-central, central, and southern areas) to estimate the $PM_{2.5}$ exposure of participants	$PM_{2.5}$, µg/m^3: Northern: 27.32 ± 4.34; North-Central: 28.65 ± 2.13; Central: 35.72 ± 3.75; Southern: 39.81 ± 2.10	average 5 years (2006-2011)	Whole blood samples	Multiple linear regression	Sex, age, alcohol drinking, exercise, BMI, exposure to SHS, and *cell type composition*

Tantoh, 2019	$PM_{2.5}$ statistics obtained from the Air Quality Monitoring Database	$PM_{2.5}$, µg /m^3 (year): Northern: 27.43 ± 5.03 (2006); 28.69 ± 5.39 (2007); 27.33 ± 5.48 (2008), 25.96 ± 5.32 (2009); 25.98 ± 5.37 (2010); 26.61 ± 5.30 (2011); Central/southern: 37.69 ± 3.59 (2006); 38.03 ± 4.05 (2007); 37.48 ± 4.59 (2008); 38.44 ± 3.67 (2009); 35.46 ± 3.14 (2010); 36.75 ± 4.97 (2011).	average 5 years (2006-2011)	Whole blood samples	Multiple linear regression	Age, sex, second-hand smoke, exercise, drinking, body fat, BMI, waist-hip ratio, asthma, and emphysema, and *cell-type composition*
Su, 2020	$PM_{2.5}$ concentrations from air monitoring stations where participants lived for at least three months	$PM_{2.5}$, µg /m^3: 26.53 (northern), 30.06 (north-central), 36.91 (central), 40.68 (southern areas)	5 years	Whole blood samples	Linear regression model	Sex, age, BMI, cigarette smoking, alcohol drinking, exposure to second-hand smoke, and *cell-type heterogeneity*
de F.C. Lichtenfels, 2018	Estimated with land-use regression models and the annual average exposure to NO_2, PM_{10} and $PM_{2.5}$	NO_2, µg /m^3: 16.3 ± 3.2, PM_{10}, µg /m^3: 24.1 ± 0.5, $PM_{2.5}$, µg /m^3: 15.5 ± 0.2	annual average (2007-2013)	Whole blood samples	Linear regression models	Sex, age, BMI, current smoking, pack-years, *technical variance*, and *blood cell composition*.
Eze, 2020	NO_2 and $PM_{2.5}$ were modelled at participants' residences	NO_2, µg/m^3 (IQR): SAP2:20.2 (14), SAP3: 16.7 (10), $PM_{2.5}$, µg/m^3 (IQR): SAP2:14.3 (5), SAP3: 12.9 (2)	1 year average	Whole blood samples	Linear mixed-effects regressions	Age, sex, education, smoking status, pack-years, passive smoking, fruit, vegetable, and alcohol intake, study area, SEP, greenness index, survey, nested study, asthma, Lden truncation indicator, and *Houseman estimates of leukocyte*

						composition, BMI, physical activity
Fiorito, 2018	Land Use Regression (LUR) models were developed and used to estimate air pollution concentrations at participants' residences	NO_2, µg/m^3: CCVD: 50.1 (15.6); Control: 49.5 (16) NOx, µg/m^3: CCVD: 96.1 (36.2); Control: 93.6 (35.6) $PM_{2.5}$, µg/m^3: CCVD: 21.9 (1.8); Control: 21.7 (1.9)	-	Blood samples	Linear regression models	Age, gender, smoking habits, centre, season, and year of recruitment, *white blood cell (WBC) percentages*
Gondalia, 2019	PM exposure estimated based on US EPA Air Quality System (AQS) monitoring data for PM_{10} (since 1987) and $PM_{2.5}$ (since 1999).	-	1 year	Peripheral blood leukocytes	Linear mixed models	Age (years) at blood draw, education, smoking status, alcohol use, physical activity, body mass index, neighbourhood socioeconomic status, mean temperature, mean dew point, mean barometric pressure, season, ten principal components (PCs) for genetic ancestry, *leukocyte proportions*, and *technical covariates*.
Honkova, 2022	Stationary air pollution monitoring of $PM_{2.5}$, B[a]P and NO_2	$PM_{2.5}$, µg/m^3 (2019): Ostrava: 17.4 ± 14.4; Prague: 12.3 ± 9.9; CB: 12.8 ± 10.4 B[a]P, ng/m^3 (2019): Ostrava: 2.0 ± 0.6; Prague: 0.7 ± 0.2; CB: 1.2 ± 0.4 NO_2, µg/m^3 (2019): Ostrava: 15.2 ± 13.5; Prague: 28.6 ± 27.5; CB: 12.9 ± 6.9	Prague: 13.9 ± 7.9, Ostrava: 11.8 ± 6.7; Ceske Budejovice: 10.2 ± 7.5	Blood samples	-	-

Liu, 2021	Measured 10 mono-hydroxylated PAHs (OH-PAHs) in urine	Total OH-PAHs ($\times 10^{-2}$), µmol/mmol creatinine, Median (25th-75th): WHZH-Wuhan: 2.30 (1.81-2.86); WHZH-Zhuhai: 2.36 (1.78-3.65); SY: 0.92 (0.63-1.68); COW: 13.82 (10.51-18.89)	COW panel: ≥ 5 years	Whole blood samples	Linear regression analyses	Age, gender, body mass index, drinking, *blood cell counts* (neutrophils, lymphocytes, and intermediate cells), and surrogate variables
Messingschlager, 2023	Measured photometrically with the DustTrack Aerosol Monitor	PM_{10}, µg/m^3: Simmerath: 16.5 ± 12.3; Stuttgart: 53.8 ± 24.7	> 1 year	Blood samples	-	Age, sex and *cell lineage proportions*
Mu, 2023	Personal $PM_{2.5}$ concentration on the same day of blood sampling was evaluated using the model as: Cp=1.02×Ca +1.37×Smo+14.76×PS −1.95×RH−9.55×(PRE −P0)−6.48×T−26.86× WS+340.01	PM_{10}, µg/m^3, (Q1-Q4): ≤70.6 - >175	-	Blood sample	Linear mixed models.	Age, gender, BMI, passive smoking (yes/no), drinking status (current/ former/never), visit year, *blood cell proportions* (CD8T, CD4T, NK cells, B cells and monocytes), *batch* (plate number and array)
Sayols-Baixeras, 2019	Used the ESCAPE protocol to assess long-term exposure to air pollution	PM_{10}, µg /m^3 (IQR): REGICOR: 29.9 (27.5 - 32.7); EPIC-Italy: 46.91 (38.24 - 53.23). $PM_{2.5}$, µg /m^3 (IQR): REGICOR: 15.0 (13.5 - 15.9); EPIC-Italy: 30.95 (26.74 - 32.73) NO_X, µg /m^3(IQR): REGICOR: 61.7 (42.5 - 72.8); EPIC-Italy: 92.83(34.46 - 131.00) NO_2, µg /m^3 (IQR): REGICOR: 36.9 (24.3 - 42.6); EPIC-Italy: 50.00 (22.26 - 67.92)	10 years	Whole blood samples	Linear regression model	Age, sex, smoking exposure,*cell composition*, and surrogate variables (i.e., traffic proximity markers)

White, 2019	Estimated by a cross-validated universal kriging models using US Environmental Protection Agency (EPA) Air Quality System (AQS) monitoring data from 2006 ($PM_{2.5}$ and NO_2) and 2000 (PM_{10})	$PM_{2.5}$, µg/m^3: 8.8, PM_{10}, µg/m^3: 21.9 NO_2, ppb: 9.9	1 year	Blood samples	Linear regression models	Age at baseline, education, smoking status, hormone therapy uses, future breast cancer case status), as well as *blood cell composition*
Curtis, 2021	PCB levels were measured in the serum	PCB, ppb: 74 ±2.55	-	Whole blood samples	Linear regression models	Age, sex, and current lipid levels
Pittman, 2020	Serum PCBs	PCBs, ppb: 516	29.6 ± 0.9	Whole blood samples	Multivariable linear regression	Age, race, sex, smoking, total lipids, and *six blood cell-type percentages*
Calderón-Garcidueñas, 2020	Cumulative $PM_{2.5}$ exposures were measured with the closest monitoring station of "Parque O'Higgins"	$PM_{2.5}$, µg/m^3: Control: <100, Exposed: 769	all their lives	Post-mortem frontal white matter sample	-	-
**Occupational air pollution exposure**						
Alegría-Torres, 2013	Urinary levels of 1-Hydroxypyrene (1-OHP) represent a fairly valid and trustworthy index of individual exposure to ambient PAHs	1-OHP, µg/g creatinine: 0.18 (range 0.023-1.11)	-	Blood sample	Linear regression models	Smoking status, usual alcohol drinking, current medication, age, and average number of cigarettes smoked

Barbosa, 2019	Monitored personal during work	FA, ppm (Q1-Q3): group A (FA< 0.01 ppm): 0.013 (0.01–0.02), group B (0.03 ppm< FA< 0.06 ppm): 0.035 (0.02–0.06), group C (0.08 ppm < FA< 0.24 ppm): 0.07 (0.05–0.17)	group A: Median = 5.5 years, group B: Median = 6 years, group C: Median = 6 years	Blood sample	Spearman correlation	Age, sex, alcohol consumption, and smoking habits
Ghosh, 2017	Measured by chemical vapor deposition (CVD) method	elemental carbon, µg/m^3: Exposed: 4.6 and 42.6	4.25 ± 2.40	Whole blood samples	Logistic regression analysis	Age, gender, smoking habit, and alcohol consumption
Issah, 2022	Personal PM was collected simultaneously every minute from all participants using a battery-operated 5-channel optical particle counter	$PM_{2.5}$, µg/m^3, median (IQR): e-waste workers: 77.32 (34.08); Control: 34.88 (16.55) PM_{10}, µg/m^3, median (IQR): e-waste workers: 210.21 (93.32); Control: 121.92 (82.93)	> 6 months	Whole blood samples	Ordinary least square (OLS) regression models	Indoor use of biomass fuel for cooking, alcohol consumption, age, smoking status, and BMI
Kile, 2013	Monitored personal $PM_{2.5}$ exposure over the duration of the work shift	m$PM_{2.5}$, µg /m^3: 0.52 (1.34) based on 54 observation days	6.8 years	Whole blood samples	Linear mixed effect models	Currently smoking (yes), *white blood cell count*, and age
Munnia, 2023	Airborne levels of benzo(a)pyrene [B(a)P], carbon monoxide, and tropospheric O_3 were determined by personal exposure	B(a)P, ng/m^3: traffic policemen: 4.2 ± 2.6 (0.5-13.9); Control: 0.2 ± 0.3 (0.03-1.1) CO, mg/m^3: traffic policemen: 8.7 ± 4.2 (1-28); Control: 1.1 ± 0.4 (1-13)	> 1 year	Peripheral blood samples	Multivariate linear regression models	Age (continuous), gender, and smoking habit (smokers and non-smokers)

	biomonitoring or by fixed monitoring stations	O_3, µg/m3: traffic policemen: 44.2 ± 23.5 (11-101); Control: -				
Guo, 2022	Gas chromatography-mass spectrometry (GC/MS) was employed to determine the concentrations of 16 prior PAH recommended by Environmental Protection Agency (EPA)	ΣPAH, ng/m^3: none exposed group: 55.01 (10.95, 71.26); low exposed group: 675.44 (367.95, 1777.03); high exposed group: 2152.01 (498.40, 6702.25)	-	Plasma samples	Multiple linear regression models	Sex, age, BMI, alcohol use, pack-years of smoking and serum folate were used as continuous variables
Hou, 2011	Used measures of airborne levels of PM mass and working hours to estimate individual exposures by a GRIMM1100 light-scattering dust analyzer	PM_{10}, µg /m^3: 233.42 ± 214.56; PM_1, µg /m3: 8.48 ± 6.18	> 1 year	Whole blood samples	Multivariate linear regression model	Age, body mass index (BMI), smoking and *percent granulocytes in the differential blood count*
Hou, 2014	Personal $PM_{2.5}$ exposure was measured during the eight hours of work	$PM_{2.5}$, µg /m^3: office workers: 94.6 ± 64.9; truck drivers: 126.8 ± 68.8	> 2 years	Peripheral blood samples	Generalized estimating equations (GEE) models	Occupation, $PM_{2.5}$, measure day, age, sex, BMI, number of cigarettes smoked during examination time and usage of central heating
Hu, 2018	Monitoring of hazardous substances in the air outlined by the national standard in China (GBZ159-2004)	Blood Cr, mg/m3, median (IQR): Exposed: 6.4 (7.2); Control: 3.9 (1.5)	5 years	Peripheral venous blood samples	Multiple linear regression model	Sex, age, BMI, smoke statues, and alcohol consumption

Tarantini, 2013	Measured during the days between time 1 and 2 using a GRIMM 1100 light-scattering dust analyzer, and individual exposure was calculated as the average of area-specific PM levels weighted by the time spent in each area	PM_{10}, µg /m^3: 233.4 ± 214.6; PM_1, µg /m^3: 8.5 ± 6.2	2 years	Blood samples	Linear mixed-effect model	Age, body mass index, current smoking status (yes/no), NSAID, *% monocytes*
Silva, 2019	$PM_{2.5}$ emitted in the construction environment was collected using a large-volume sampler with quartz-fiber filters; Concentrations of PAHs and nitro-PAHs were determined by gas chromatography/mass spectrometry	$PM_{2.5}$, µg /m^3: 51 - 841; PAHs, ng/m^3: 0.01 - 0.6	10 years	Blood samples	Multiple linear regression	Previous smoking habits, exposure to passive smoking, age and alcohol consumption
Xu, 2017	Used respirable dust as the indicator of exposure to particles from welding fumes	Respirable dust, mg/m^3, median (5-95 percentile): Welder: 1.1 (0.2-8.4); Control: 0.1 (0.0-0.1)	-	Peripheral blood samples	Linear regression models	Age, BMI, previous smoking years, smokeless tobacco “snus” status and current residence
Zhang, 2015	urinary 1-hydroxypyrene (1-OH-Py) levels were determined by high-performance liquid chromatography	1-OH-Py, mmol/mol Cr: Exposed: 0.46 ± 0.12; Controls: 0.17 ± 0.06	Exposed: 17.81 ± 7.62; Control: 19.15 ± 6.98	Whole blood samples	-	-

Alhamdow, 2020	PAH (phenanthrene and fluorene) metabolites (∑OH-phenanthrene and ∑OH-fluorene) in urine	∑OH-phenanthrene, µg/g crea. (minimum-maximum): 0.27 (0.070-5.1), ∑OH-fluorene, µg/g crea.: 0.64 (0.086-6.6) (PAHs metabolites)	7.17 (5.83) years	Whole blood samples	Linear regression models	Age and smoking
Jiménez-Garza, 2015	Monitored VOC levels during work	Benzene, µg /m^3: 190 ± 10 (NE);190 ± 60 (E); Ethylbenzene, µg /m^3: 240 ± 20 (NE); 250 ± 120 (E) Toluene, µg /m^3: 210 ± 20 (NE); 10800 ± 7500 €	E: 67.8±68.1 months	Whole blood samples	Linear regression models	Age, BMI, toluene exposure levels, smoking status, cumulative time of exposure and place of labour
Jiménez-Garza, 2018	Monitored the individual exposure levels of VOCs	Benzene, µg /m^3 (95%CI): LS: 50 (33 - 74), GS: 162 (103 - 255), C: 2 (1 - 3) Toluene, µg /m^3 (95%CI): LS: 32552 (1360 - 78726) GS: 481 (365 - 634) C: 8 (5 - 13) Ethylbenzene, µg /m^3 (95%CI): LS: 1969 (882 - 4396) GS: 66 (48 - 90) C: 2 (1 - 3)	LS: 117.6±127.9; GS: 76.03±90.8; C: NA (month)	Whole blood samples	Linear regression model	Sex, age, BMI, cumulative time of exposure and smoking status
Xing, 2013	Dosemeci exposure assessment method used to estimate benzene exposure	Benzene, µg/m^3 (per year): 1.13×106 (Tianjin); over 3.49×105(Shanghai)	5 years	Whole blood samples	Linear regression models	Age, sex, smoking status, alcohol drinking, body mass index (BMI) and *percentage of lymphocytes, neutrophils, and monocytes*
Zheng, 2017	Cumulative exposures calculated by summing the workplace estimates over the exposure duration	benzene, µg /m^3: >3.5×105 (per year)	20 ± 9 years	Whole blood samples	Linear regression models	Sex, age, alcohol drinking, smoking status, BMI, and *percentage of lymphocytes, neutrophils, and monocytes*

Cantone, 2011	Personal exposure was calculated as the average of work area levels weighted by the time spent in each area	PM_1, µg/m^3: (73.72-1220.17) PM_{10}, µg/m^3: (1.71-30.49)	over 3 years	Blood sample	Multivariable regression models	Age, BMI, education, pack-years, and *percent granulocytes*
Li, 2018	Urinary S-phenylmercapturic acid (SPMA) and S-benzylmercapturic acid (SBMA) were measured as the benzene exposure	SPMA, ng/g Cr., Median (5%-95%): Benzene-exposed workers: 100.12 (20.97-656.74); control workers: 55.25 (9.62-249.68) SBMA, ng/g Cr., Median (5%-95%): Benzene-exposed workers: 2.53 (0.87-11.01); control workers: 2.37 (0.76-10.84)	22.87 ± 6.40	Blood samples	Multivariate linear models	Age, BMI and urinary S-phenylmercapturic acid (SPMA) and S-benzylmercapturic acid (SBMA)
Yang, 2018	Urinary 1-hydroxypyrene was measured to present the PAHs exposure	1-hydroxypyrene, µg/mmol creatinine: Coke oven workers: 0.06 (0.02–0.38); Non-coke oven workers: 0.04 (0.02–0.14).	Coke oven workers: 20 (1-31) years; non-coke oven workers: 22 (4-35) years	Blood samples	Multivariate logistic regression models	Gender, age, heating mode, smoking, drinking, 2-hydroxynaphthalene, 2-hydroxyfluorene, and 9-hydroxyphenanthrene

Note: Technical factors (i.e., batch, chip or cell composition effects) were indicated in italics. BMI, body mass index; FVC, forced vital capacity; DLCO, diffusion capacity of the lung for carbon monoxide; PFF, Pulmonary Fibrosis Foundation Patient Registry sites; NSAIDs, non-steroidal anti-inflammatory drugs.

Table S6. Epigenetic alterations assessment techniques used among included studies.

Methylation measurement technique	Global DNA methylation	Candidate-gene	EWAS	Histone modification
**Pyrosequencing-based approaches**				
Pyrosequencing	[1-6]	[7-17]		
**Array-based approaches**				
Infinium HumanMethylation450 BeadChip	[6,18-20]		[18-28]	
Infinium® MethylationEPIC BeadChipEPIC array		[29-32]	[33-36]	
**High-throughput sequencing-based**				
Whole-Genome Bisulfite Sequencing (WGBS)			[37,38]	
**Analytical chemistry-based approaches**				
Luminometric Methylation Assay (LUMA)	[39]			
Enzyme-linked immunosorbent assay (ELISA)	[40-42]			[43,44]
Methylation-specific quantitative polymerase chain reaction (MS-qPCR)		[45,46]		
High-pressure liquid chromatography with diode array detection (HPLC-DAD)	[47]			
Liquid chromatography tandem-mass spectrometry (LC-MS/MS)	[48]	[48]		
Matrix-assisted laser desorption ionization-time of flight mass spectrometry (MALDI-TOF MS)		[49-51]		
**Antibody-based enrichment approaches**				
Chromatin Immunoprecipitation (ChIP)				[52]

Table S7. Quality assessment-ROBINS-E tool.

First author, year	Exposure Assessment	Misclassification and measurement error of outcome	Control of confounding factors		Selection Bias/loss to follow up	Risk of bias							
			Socioeconomic control	Seasons, temperature and smoking status control		A	B	C	D	E	F	G	Overall
Alegría-Torres, 2013	Urinary levels of 1-Hydroxypyrene (1-OHP) represent a fairly valid and trustworthy index of individual exposure to ambient PAHs	In-person assessment supplemented with Pyrosequencing	Individual level adjustment	No control of seasons and temperature	Cross - sectional study	Some	Low	Low	Some	Low	Low	Low	Some
Barbosa, 2019	HPLC-DAD equipment monitored personal air pollution during work in the metropolitan region of Porto Alegre	Relative content of 5-methyldeoxy-cytidine (5mdC) assessment by high-pressure liquid chromatography with diode array detection (HPLC-DAD)	Individual level adjustment	No control of seasons and temperature	Cross - sectional study	Some	Some	Low	Some	High	Low	Low	High
Calderón-Garcidueñas, 2020	Cumulative $PM_{2.5}$ exposures were measured based on closest monitoring station of “Parque O'Higgins” assigned to residence	In-person assessment supplemented with Chromatin Immunoprecipitation (ChIP)	Area level adjustment	No control of seasons, temperature and smoking	No discussion of loss to follow-up	High	Low	High	Some	Some	Low	Low	High

Callahan, 2018	Assessed from New York State Department of Environmental Conservation air monitors assigned at residence level	In-person assessment supplemented with Pyrosequencing	Individual level adjustment	No control of seasons and temperature	Case - control study	Some	Low	Low	Some	Some	Low	Low	Some
Cantone, 2011	Personal exposure was calculated as the average of work area levels weighted by the time spent in each area	In-person assessment supplemented with Enzyme-linked immunosorbent assay (ELISA)	Individual level adjustment	No control of seasons and temperature	Cross - sectional study	Some	Some	Low	Low	Low	Some	Low	Some
Cantone, 2020	Personal exposure monitored by the Regional Environmental Protection Agency	In-person assessment supplemented with Pyrosequencing	Individual level adjustment	No control of seasons and temperature	Cross - sectional study	Some	Low	Low	Low	Some	Some	Low	Some
Cheng, 2022	Individual 24 state-owned air concentration was identified by the monitoring data assigned to residential address	In-person assessment supplemented with Enzyme-linked immunosorbent assay (ELISA)	Individual level adjustment	Control of seasons, temperature and smoking	Cross - sectional study	Low	Low	Low	Low	Some	Low	Low	Some

Chi, 2016	Monitored by the U.S. Environmental Protection Agency-operated Air Quality System monitors using spatiotemporal models assigned at residence level	In-person assessment supplemented with Illumina's Infinium HumanMethylation 450 BeadChip	Individual level adjustment	No control of seasons and temperature	Cross - sectional study	Low	Low	Low	Low	Low	Low	Low	Low
Chi, 2022	Monitored by the U.S. Environmental Protection Agency-operated Air Quality System monitors using spatiotemporal models assigned at residence level	In-person assessment supplemented with Illumina's Infinium HumanMethylation 450 BeadChip	Individual level adjustment	No control of seasons and temperature	Follow-up visits over a 10-year period since baseline	Low	Low	Low	Low	Low	Low	Low	Low
Chou, 2020	Estimated participants' exposure from Air Quality Monitoring Database (AQMD); assigned 2006–2011 residential addresses	In-person assessment supplemented with Illumina Infinium 850K MethylationEPIC BeadChip	Individual level adjustment	No control of seasons and temperature	Cross - sectional study	Some	Low	Low	Low	Some	Low	Low	Some
de F.C. Lichtenfels, 2018	Estimated with land-use regression models and the annual average exposure to NO_2, PM_{10} and $PM_{2.5}$ assigned at home addresses	In-person assessment supplemented with Illumina® HumanMethylation 450K BeadChip	Individual level adjustment	No control of seasons and temperature	Cross - sectional study	Some	Low	Low	Low	High	Low	Low	High

Eze, 2020	Personal exposures were modelled at participants' residences using a hybrid model	In-person assessment supplemented with Illumina Infinium 450K BeadChip	Individual level adjustment	No control of seasons and temperature	Cross - sectional study	Low	Low	Low	Low	Low	Low	Low	Low
Fiorito, 2018	Land Use Regression (LUR) models were developed and used to estimate air pollution concentrations at residences level	In-person assessment supplemented with Infinium HumanMethylation 450 BeadChip	Individual level adjustment	Control of seasons and smoking	Case - control study	Some	Low	Low	Low	Low	Low	Low	Some
Ghosh, 2017	Measured by chemical vapor deposition (CVD) method based on exposure assignment	In-person assessment supplemented with bisulfite pyrosequencing	Individual level adjustment	No control of seasons and temperature	Cross - sectional study	Some	High	Low	Some	High	Low	Low	High
Gondalia, 2019	PM exposure estimated based on US EPA Air Quality System (AQS) monitoring data assigned to residential addresses	In-person assessment supplemented with Illumina 450K Infinium Methylation BeadChip	Individual level adjustment	Control of seasons, temperature and smoking	Cross - sectional study	Low	Low	Low	Low	Some	Low	Low	Some
Goobie, 2023	Measured from the Atmospheric Composition Analysis Group online data repository assigned at residence	In-person assessment supplemented with ELISA-based MethylFlash assay	Individual level adjustment	No control of seasons and temperature	Loss to follow -up discussed	Some	Low	High	Some	Some	Low	Low	High

Guo, 2022	Gas chromatography-mass spectrometry (GC/MS) was employed to determine the concentrations recommended by Environmental Protection Agency (EPA) assigned at workplaces	In-person assessment supplemented with Polymerase chain reaction (PCR)-Pyrosequencing	Individual level adjustment	No control of seasons and temperature	Cross - sectional study	Some	Low	Low	Some	High	Low	Low	High
Honkova, 2022	Stationary air pollution monitoring of exposure in each city	In-person assessment supplemented with Illumina Infinium 850K MethylationEPIC BeadChip	No adjustment	No control of seasons, temperature and smoking	Cross - sectional study	High	Low	Low	Some	High	Low	Low	High
Hou, 2011	Estimating individual exposures by a GRIMM1100 light-scattering dust analyser assigned at workplace	In-person assessment supplemented with Pyrosequencing	Individual level adjustment	No control of seasons and temperature	Cross - sectional study	Some	Low	Low	Low	High	Some	Low	High
Hou, 2014	Personal exposure was measured with two monitors at the same time during the hours of work	In-person assessment supplemented with Polymerase chain reaction (PCR)-Pyrosequencing	Individual level adjustment	No control of seasons and temperature	Cross - sectional study	Some	Low	Low	Some	High	Low	Low	High

Hu, 2018	Monitoring of hazardous substances in the air outlined by the national standard with occupational exposure	In-person assessment supplemented with MALDI-TOF-MS of the MassArray system	Individual level adjustment	No control of seasons and temperature	Cross - sectional study	Some	Low	Low	Some	High	Some	Low	High
Issah, 2022	Personal exposure was collected simultaneously using a battery-operated 5-channel optical particle counter at workplace during peak working hours	In-person assessment supplemented with Pyrosequencing	Individual level adjustment	No control of seasons and temperature	Cross - sectional study	Some	Some	Low	Some	High	Some	Low	High
Kile, 2013	Monitored personal exposure over the duration of the work shift assigned at participant's shoulder in the breathing zone area.	In-person assessment supplemented with Polymerase chain reaction (PCR)-Pyrosequencing	Individual level adjustment	No control of seasons and temperature	Low loss to follow-up discussed	Some	Low	Low	Low	High	Low	Low	High
Li, 2018	Urinary S-phenylmercapturic acid (SPMA) and S-benzylmercapturic acid (SBMA) were measured as the benzene exposure assigned at workplace	In-person assessment supplemented with sandwich enzyme-linked immunosorbent assay (ELISA)	Individual level adjustment	No control of seasons, temperature and smoking	Cross - sectional study	Some	Some	Low	Some	High	Some	Low	High

Liu, 2021	Measuring 10 mono-hydroxylated PAHs (OH-PAHs) in urine by Agilent 6890N + 5975B gas chromatography-mass spectrometry system	In-person assessment supplemented with Illumina 450K Infinium Methylation BeadChip	Individual level adjustment	No control of seasons, temperature and smoking	Cross - sectional study	Some	Some	Low	Low	Low	Low	Low	Some
Messingschl ager, 2023	Measuring photometrically with the DustTrack Aerosol Monitor assigned at residential address >350 local measuring stations	In-person assessment supplemented with Whole-Genome Bisulfite Sequencing (WGBS)	No adjustment	No control of seasons, temperature and smoking	Cross - sectional study	High	Low	Low	Low	Some	Low	Low	High
Mu, 2023	Evaluating (using the model as: Cp=1.02×Ca +1.37×Smo+14.76×PS−1.95×RH−9.55×(PRE−P0)−6.48×T−26.86×WS+340.01) from the nearest fixed monitoring station	In-person assessment supplemented with Illumina Infinium 850K MethylationEPIC BeadChip	Individual level adjustment	No control of seasons and temperature	Cross - sectional study	Some	Low	Low	Low	Low	Low	Low	Some
Munnia, 2023	Determining by personal exposure biomonitoring or by fixed monitoring stations assigned at area-level geocoding	In-person assessment supplemented with Polymerase chain reaction (PCR)-pyrosequencing	Individual level adjustment	No control of seasons and temperature	Cross - sectional study	Some	Low	Low	Some	High	Low	Low	High

Plusquin, 2017	Used land-use regression models to assess exposure of each participant assigned at the baseline address	In-person assessment supplemented with Illumina Infinium® HumanMethylation 450 BeadChip	Individual level adjustment	No control of seasons and temperature	Cross - sectional study	Some	Low	Low	Low	Low	Low	Low	Some
Sayols-Baixeras, 2019	Using land use regression (LUR) models to weighted average exposures based on address histories for the past 10 years	In-person assessment supplemented with HumanMethylation 450 BeadChip	Individual level adjustment	No control of seasons and temperature	Cross - sectional study	Some	Low	Low	Low	Low	Low	Low	Some
Silva, 2019	Collected using a large-volume sampler with quartz-fiber filters assigned at workplace	In-person assessment supplemented with Pyrosequencing	No adjustment	No control of seasons and temperature	Cross - sectional study	High	Some	Low	Some	High	Low	Low	High
Song, 2019	Measured urinary BPA concentration of the workers as the BPA exposure	In-person assessment supplemented with Methylation-specific quantitative polymerase chain reaction (MS-qPCR)	Individual level adjustment	No control of seasons and temperature	12% loss to follow up	Some	Some	Some	Some	High	Low	Low	High
Tantoh, 2019	Estimated from the monitors of each area (northern, north-central, central, and southern areas) (2006-2011) corresponding to the	In-person assessment supplemented with Illumina Infinium 850K MethylationEPIC BeadChip	Individual level adjustment	No control of seasons, temperature and smoking	Cross - sectional study	Some	Low	Low	Low	Some	Low	Low	Some

	participants' residence												
Tantoh, 2019	Exposure statistics obtained from the Air Quality Monitoring Database assigned at residence level	In-person assessment supplemented with Illumina Infinium 850K MethylationEPIC BeadChip	Individual level adjustment	No control of seasons and temperature	Cross - sectional study	Some	Low	Low	Low	Some	Low	Low	Some
Tao, 2014	The sum of the differences between the ages at starting and leaving each residence in which solid fuels were used as principle means for cooking or heating	In-person assessment supplemented with Luminometric Methylation Assay (LUMA)	Individual level adjustment	No control of seasons and temperature	Case - control study	Some	Some	Low	Some	Low	Low	Low	Some
Tarantini, 2013	Calculating as the average of area-specific PM levels weighted by GRIMM 1100 light-scattering dust analyzer measurement and the time spent in each workplace	In-person assessment supplemented with Pyrosequencing	Individual level adjustment	No control of seasons and temperature	Cross - sectional study	Some	Some	Low	Low	Some	Low	Low	Some

Wang, 2020	A regionalized national universal kriging model using partial least squares to estimate annual exposure concentrations for the current residences	In-person assessment supplemented with Pyrosequencing	Individual level adjustment	No control of seasons and temperature	Cross - sectional study	Some	Low	Low	Low	Low	Low	Low	Some
Wang, 2020	Derived from environmental monitoring organizations at province level	In-person assessment supplemented with Whole-Genome Bisulfite Sequencing (WGBS)	Individual level adjustment	No control of seasons and temperature	Cross - sectional study	Some	Low	Low	Low	Some	Low	Low	Some
White, 2019	Estimated by a cross-validated universal kriging models using US Environmental Protection Agency (EPA) Air Quality System (AQS) monitoring data based on participants' residential addresses	In-person assessment supplemented with Illumina HumanMethylation 450 BeadChips	Individual level adjustment	No control of seasons and temperature	Cross - sectional study	Some	Low	Low	Low	Low	Low	Low	Some

Xu, 2023	Estimated annual average exposure across the globe by combining data from satellite observations, chemical transport models, and ground-based observations assigned at 1.1 km × 1.1 km at the equator	In-person assessment supplemented with HumanMethylation 450 BeadChip array	Individual level adjustment	Control of seasons, temperature and smoking	Cross - sectional study	Low	Low	Low	Low	Low	Low	Low	Low
Xu, 2017	Used respirable dust as the indicator of exposure to particles from welding fumes based on the workers' breathing zone	In-person assessment supplemented with Polymerase chain reaction (PCR)-Pyrosequencing	Individual level adjustment	No control of seasons and temperature	Cross - sectional study	Some	Some	Low	Some	High	Low	Low	High
Yadav, 2021	Estimated by distance of residential area from the main road	In-person assessment supplemented with Enzyme-linked immunosorbent assay (ELISA)	Individual level adjustment	No control of seasons and temperature	Cross - sectional study	Some	Some	Low	Some	Some	Low	Low	Some
Zhang, 2015	Determined by high-performance liquid chromatography	In-person assessment supplemented with Methylation-specific quantitative polymerase chain reaction (MS-qPCR)	No adjustment	No control of seasons, temperature and smoking	Cross - sectional study	High	Some	Low	Some	Low	Low	Low	High

Alhamdow, 2020	Estimated by PAH (phenanthrene and fluorene) metabolites (∑OH-phenanthrene and ∑OH-fluorene) in urine	In-person assessment supplemented with Polymerase chain reaction (PCR)-Pyrosequencing	Individual level adjustment	No control of seasons and temperature	Cross - sectional study	Some	Some	Low	Some	Low	Low	Low	Some
Curtis, 2021	PCB levels were measured in the serum	In-person assessment supplemented with MethylationEPIC Beadchip	Individual level adjustment	No control of seasons, temperature and smoking	Cross - sectional study	Some	Some	Low	Some	Low	Low	Low	Some
Jiménez-Garza, 2015	Monitored volatile organic compounds (VOC) levels during work	In-person assessment supplemented with Polymerase chain reaction (PCR)-Pyrosequencing	Individual level adjustment	No control of seasons and temperature	Cross - sectional study	Some	Some	Low	Some	High	Low	Low	High
Jiménez-Garza, 2018	Monitored the individual exposure levels of VOCs at the breathing zone level	In-person assessment supplemented with Polymerase chain reaction (PCR)-Pyrosequencing	Individual level adjustment	No control of seasons and temperature	No information of loss to follow-up	Some	Some	High	Some	High	Low	Low	High
Pittman, 2020	Serum Polychlorinated biphenyl (PCBs)	In-person assessment supplemented with Illumina Infinium 850K MethylationEPIC BeadChip	Individual level adjustment	No control of seasons and temperature	Cross - sectional study	Some	Some	Low	Low	Low	Low	Low	Some
Su, 2020	Exposure concentrations from air monitoring	In-person assessment supplemented with Infinium®	Individual level adjustment	No control of seasons and temperature	Cross - sectional study	Some	Low	Low	Low	Some	Low	Low	Some

	stations where participants lived	MethylationEPIC BeadChipEPIC array											
Xing, 2013	Estimating benzene exposure by Dosemeci exposure assessment method assigned at workplace	In-person assessment supplemented with MALDI-TOF MS	Individual level adjustment	No control of seasons and temperature	Cross - sectional study	Some	Some	Low	Low	High	Low	Low	High
Zheng, 2017	Cumulative exposures calculated by summing the workplace estimates over the exposure duration based on monitoring data	In-person assessment supplemented with MALDI-TOF MS	Individual level adjustment	No control of seasons and temperature	No information of loss to follow-up	Some	Low	High	Low	High	Low	Low	High
Yang, 2018	The ΣOH-PAHs concentration was calculated by the sum of four measured PAHs metabolites	In-person assessment supplemented with Polymerase chain reaction (PCR)-Pyrosequencing	Individual level adjustment	No control of seasons and temperature	No information of loss to follow-up	Some	Some	Low	Some	Low	Low	Low	Some

Table S8. Genes methylation investigated in candidate-gene studies.

First author, year	Type of pollutant	Gene	Regression coefficient (β / OR), Adjusted	Low Confidence Interval, 95%	High Confidence Interval, 95%	SE
**Ambient air pollution exposure**						
Cantone, 2020	$PM_{2.5}$	*ARNTL*	-0.11	-0.27	0.05	0.08
		CLOCK	-0.04	-0.16	0.09	0.06
		NPAS2	-0.20	-0.20	-0.20	-0.20
		CRY1	0.04	0.04	0.12	0.04
		CRY2	0.12	-0.06	-0.06	0.12
		PER1	-0.03	0.05	0.08	0.05
		PER2	0.08	-2.09	0.23	0.08
		PER3	0.38	0.77	2.00	0.77
Chou, 2020	$PM_{2.5}$	*DLEC1*	0.000114	-	-	-
Su, 2020	$PM_{2.5}$	*SOX2*	0.00022	0.00011	0.00032	0.00005
Tantoh, 2019	$PM_{2.5}$	*AHRR*	-0.00115	-	-	-
Tantoh, 2019	$PM_{2.5}$	*SOX2*	0.00042	0.00025	0.00059	0.00009
Callahan, 2018	TSP	*SFN**	1.00	-	-	-
		*SFN***	1.08	0.67	1.75	-
		*SCGB3A1**	1.00	-	-	-
		*SCGB3A1***	0.89	0.52	1.52	-
		*RARB**	1.00	-	-	-
		*RARB***	0.93	0.58	1.47	-
		*GSTP1**	1.00	-	-	-
		*GSTP1***	0.79	0.50	1.26	-
		*CDKN2A**	1.00	-	-	-
		*CDKN2A***	0.99	0.60	1.66	-
		*CCND2**	1.00	-	-	-
		*CCND2***	0.48	0.26	0.89	-
		*BRCA1**	1.00	-	-	-
		*BRCA1***	1.01	0.66	1.55	-
		*FHIT**	1.00	-	-	-
		*FHIT***	1.05	0.52	2.14	-
		*SYK**	1.00	-	-	-
		*SYK***	0.99	0.64	1.54	-
Song, 2019	BPA	*ACHE*	0.046	0.000	0.092	-
**Occupational air pollution exposure**						
Cantone, 2011	PM_{10}	*H3K4me2*	0.05	-0.04	0.14	-
		H3K9ac	0.00	-0.17	0.17	-
Hou, 2011	PM_{10}	*APC*	0.27	0.13	0.40	-
		p16	0.01	-0.17	0.20	-
		p53	0.00	-0.12	0.12	-
		RASSF1A	0.03	-0.13	0.19	-
Tarantini, 2013	PM_{10}	*NOS3*	-0.20	-0.40	-0.03	-
		EDN1	0.02	-0.10	0.10	-
Cantone, 2011	PM_1	*H3K4me2*	0.07	-0.06	0.21	-
		H3K9ac	0.08	-0.17	0.32	-
Hou, 2011	PM_1	*APC*	0.23	0.09	0.38	-
		p16	0.02	-0.17	0.21	-
		p53	-0.06	-0.16	0.05	-
		RASSF1A	0.04	-0.14	0.21	-
Tarantini, 2013	PM_1	*NOS3*	-0.8	-1.40	-0.10	-
		EDN1	0.30	-0.20	0.70	-

Alegría-Torres, 2013	PAHs	*IL-12*	-1.57	-2.90	-0.23	-
		p53	-2.70	-5.46	0.06	-
		TNF-a	-3.90	-8.28	0.48	-
		Alu	-0.55	-1.25	0.16	-
		IFN-γ	-0.43	-16.45	15.59	-
		IL-6	0.22	-9.19	9.63	-
		LINE-1	-0.49	-4.74	3.76	-
Alhamdow, 2020	PAHs	*F2RL3*	-2.7	-3.9	-1.5	
		AHRR	-7.1	-9.6	-4.7	
Silva, 2019	$PM_{2.5}$; PAHs	*CDKN2A*	1.14	-	-	0.61
		MLH1	1.86	-		0.45
		APC	1.05	-	-	0.50
		LINE-1	-1.12	-	-	0.53
Li, 2018	Benzene	*H3K4me3*	0.11	0.007	0.214	-
		H3K36me3	-0.038	-0.155	0.078	-
		H3K79me2	0.008	-0.11	0.126	-
Xing, 2013	Benzene	*BLM*	0.11	-	-	0.06
		CYP1AW1	0.30	-	-	0.53
		EPHX1	0.03	-	-	0.07
		ERCC3	0.27	-	-	0.13
		NQO1	0.00	-	-	0.06
		NUDT1	0.16	-	-	0.17
		p15	0.02	-	-	0.11
		p16	0.00	-	-	0.05
		RAD51	-0.13	-	-	0.12
		TP53	0.01	-	-	0.06
		WRAP53	-0.06	-	-	0.04
Xu, 2017	RD	*MT-TF*	-1.50	-2.50	-0.48	-

PAHs=Polycyclic Aromatic Hydrocarbons; BPA=bisphenol A; TSP=Total Suspended Particulates; RD=Respirable dust; *= total suspended particulates=134 μg/m^3; **= total suspended particulates>134 μg/m^3.

Table S9. Differentially methylated sites (CpGs) identified in included studies.

First author, year	Type of pollutant	Probe	Mapped gene	Location in gene	Relation to CpG island	Regression coefficient (β / OR), Adjusted	Low Confidence Interval, 95%	High Confidence Interval, 95%	SE
$PM_{2.5}$									
Chi, 2016	$PM_{2.5}$	cg20455854	*ANKHD1*	-	-	0.139	0.074	0.203	-
		cg07855639	*LGALS2*	-	-	0.081	0.043	0.120	-
		cg07598385	*ANKRD11*	-	-	0.108	0.056	0.160	-
		cg17360854	*BAZ2B*	-	-	0.081	0.042	0.120	-
		cg23599683	*PPIE*	-	-	-0.057	-0.085	-0.029	-
Chi, 2022	$PM_{2.5}$	cg05926640	*TOMM20*	gene body	-	0.049	0.032	0.067	-
		cg04310517	*KREMEN2*	gene body	-	0.052	0.033	0.072	-
		cg09509909	*FGFBP3*	gene body	-	0.079	0.049	0.11	-
Eze, 2020	$PM_{2.5}$	cg26704043	*FARS2*	5' UTR	-	0.014	-	-	0.003
		cg05157625	*RIN3*	Body	-	0.021	-	-	0.004
		cg20099458	*WIPI2*	3'UTR	-	0.014	-	-	0.003
		cg06587257	*ACCN2*	5' UTR	-	0.023	-	-	0.005
		cg14531665	*SPIN1*	Body	-	0.011	-	-	0.003
		cg06526020	*NUDT3*	Body	-	0.028	-	-	0.006
		cg21058520	-	-	-	0.004	-	-	0.001
		cg16259904	*LRRC27*	5' UTR	-	0.027	-	-	0.006
		cg12770741	*NXN*	TSS1500	-	0.018	-	-	0.004
		cg26750893	-	-	-	0.016	-	-	0.004
Gondalia, 2019	$PM_{2.5}$	cg01948201	*HSPC159*	Body	Island	-6.41E-04	-	-	1.29E-04
		cg07316313	*LIF*	Body	Island	-6.73E-04	-	-	1.38E-04
		cg24988255	*HOXA11AS; HOXA11*	Body; TSS1500	Island	-7.74E-04	-	-	1.61E-04
		cg15201877	*PTGER3*	1st Exon; Body	Island	-6.54E-04	-	-	1.41E-04
		cg02573089	*PRR11; SKA2; SKA2*	TSS200; TSS1500; TSS1500	S_Shore	1.22E-04	-	-	2.64E-05
		cg15232798	*WWC1*	Body	Island	-4.70E-04	-	-	1.02E-04
		cg22453435	*FLJ13197; KLF3*	TSS1500; 5'UTR	S_Shore	6.65E-04	-	-	1.46E-04
		cg21632975	*NOVA2*	Body	Island	-7.03E-04	-	-	1.56E-04
Mu, 2023	$PM_{2.5}$	cg10314909	*MMEL1*	Body	N_Shelf	-0.004	-	-	-
		cg24821877	*PI4KB*	5′UTR	N_Shore	0.002	-	-	-
		cg08968107	*COL9A1*	TSS200	OpenSea	-0.003	-	-	-
		cg18362281	*DNAJB6*	Body	Island	-0.003	-	-	-
		cg24862131	-	-	S_Shelf	0.002	-	-	-
		cg24663971	-	-	OpenSea	-0.003	-	-	-
		cg23530876	*LINC01314*	Body	N_Shore	0.002	-	-	-
		cg11149743	*HOXB7*	TSS200	Island	0.004	-	-	-
		cg17834632	*METRNL*	Body	S_Shore	-0.003	-	-	-
		cg10781276	-	-	S_Shore	0.002	-	-	-
Plusquin, 2017	$PM_{2.5}$	cg12575202	-	-	-	-0.467	-	-	0.080
		cg08630381	-	-	Island	0.461	-	-	0.073
		cg17629796	-	-	-	-0.563	-	-	0.094
		cg07084345	-	-	-	-0.563	-	-	0.075
		cg04319606	*C2orf70*	TSS200	Island	0.261	-	-	0.068
		cg09568355	-	-	Island	0.261	-	-	0.050
		cg03513315	*PES1*	TSS1500	Island	0.307	-	-	0.058
		cg25489413	*ZMIZ2*	TSS1500	-	-0.365	-	-	0.068
		cg00005622	-	-	North shore	-0.398	-	-	0.064
		cg23890774	-	-	Island	0.078	-	-	0.014

Sayols-Baixeras, 2019	$PM_{2.5}$	cg10893043	*Intergenic*	-	-	0.043	-	-	0.011
		cg05088605	*LRRC45*	-	-	-0.042	-	-	0.011
		cg16560256	*PXK*	-	-	-0.044	-	-	0.012
Tantoh, 2019	$PM_{2.5}$	cg05575921	*AHRR*	-	-	-0.001	-	-	-
Xu, 2023	$PM_{2.5}$	cg10778288	-	IGR	island	0.004	-	-	0.001
		cg12764201	*CORT*	1st Exon	opensea	-0.011	-	-	0.002
		cg24741703	-	IGR	opensea	-0.005	-	-	0.001
		cg09326880	*C3orf39*	TSS200	island	0.020	-	-	0.000
		cg09447811	*CASR*	5′UTR	opensea	-0.115	-	-	0.002
		cg16249932	*MAEA*	Body	opensea	-0.005	-	-	0.001
		cg23866166	-	IGR	shore	-0.003	-	-	0.001
		cg17796333	-	IGR	opensea	-0.005	-	-	0.001
		cg11412046	*ENPP1*	Body	opensea	-0.004	-	-	0.001
		cg01676795	*POR*	Body	opensea	-0.115	-	-	0.002
		cg22659370	*PEG10*	5′UTR	shore	-0.003	-	-	0.001
		cg11829680	*EXT1*	Body	opensea	-0.013	-	-	0.002
		cg14979593	*PGAP2*	Body	shore	-0.006	-	-	0.001
		cg17287172	*MYO7A*	Body	shore	-0.006	-	-	0.001
		cg03327443	-	IGR	shelf	-0.007	-	-	0.001
		cg03631596	*C13orf37*	TSS200	island	-0.001	-	-	0.000
		cg05397010	*PLA2G4F*	Body	opensea	-0.006	-	-	0.001
		cg12281446	*SNAP23*	TSS1500	shelf	-0.004	-	-	0.001
		cg01985177	*CRYM*	TSS200	island	-0.002	-	-	0.000
		cg07021331	*PLCG2*	5′UTR	opensea	-0.002	-	-	0.000
		cg02957270	*SKAP1*	TSS1500	shore	0.010	-	-	0.002
		cg08200770	*TBCD*	Body	opensea	-0.008	-	-	0.001
		cg22994198	*ADAMTSL5*	Body	shore	0.016	-	-	0.003
		cg06751603	*SMARCA4*	Body	island	-0.003	-	-	0.001
		cg14724750	*CTU1*	TSS200	island	-0.003	-	-	0.001
		cg05054998	*C21orf70*	Body	island	-0.002	-	-	0.000
		cg26058289	*GRAMD4*	Body	island	-0.002	-	-	0.000
PM_{10}									
Gondalia, 2019	PM_{10}	cg16180082	*TAGAP*	TSS1500		-9.85E-04	-	-	2.01E-04
		cg25330361	*CYP2E1*	Body	Island	9.91E-04	-	-	2.08E-04
		cg09754549	*LOC100130274*	TSS1500	Island	1.74E-03	-	-	3.66E-04
		cg14641231	-	-	S_Shore	-6.35E-04	-	-	1.39E-04
		cg21187669	*ATPAF2*	Body	-	-7.29E-04	-	-	1.60E-04
		cg21015808	-	-	-	1.21E-03	-	-	2.72E-04
		cg27531587	*C20orf160*	3'UTR	Island	-1.10E-03	-	-	2.47E-04
Plusquin, 2017	PM_{10}	cg17629796	-	-	-	-0.386	-	-	0.073
		cg03025825	*SMG6*	Body	Island	-0.396	-	-	0.075
		cg03513315	*PES1*	TSS1500	-	0.223	-	-	0.042
		cg21232615	*C9orf11*	Body	-	-0.345	-	-	0.061
NO_2									
de F.C. Lichtenfels, 2018	NO_2	cg04908668	*PSMB9*	Body	S_Shore	-0.012	-	-	0.002
		cg14938677	*ARF5*	3'UTR	S_Shelf	0.023	-	-	0.004
		cg00344801	*TTC38*	Body	Island	-0.028	-	-	0.005
		cg18379295	*GNG2*	TSS1500	OpenSea	0.020	-	-	0.004
		cg25769469	*PTCD2*	Body	OpenSea	0.035	-	-	0.006
		cg02234653	*AP1S3*	Body	OpenSea	-0.017	-	-	0.003
		cg08500171	*BAT2*	Body	S_Shore	0.023	-	-	0.004
Eze, 2020	NO_2	cg04337651	*ASB1*	Body	-	0.003	-	-	0.001
		cg18776472	*ERCC6*	Body	-	-0.001	-	-	0.000
		cg18601596	*KCNK16*	Body	-	0.006	-	-	0.001
		cg12392998	*NPLOC4*	Body	-	-0.002	-	-	0.000
		cg16550606	*RCBTB1*	TSS1500	-	0.004	-	-	0.001

		cg25266109	*ZNF44*	Body	-	0.000	-	-	0.000
		cg01746514	*LRRC16B*	TSS1500	-	-0.001	-	-	0.000
		cg15811902	*SNUPN*	5' UTR	-	-0.002	-	-	0.001
		cg26898336	*TEKT3*	5' UTR	-	0.002	-	-	0.001
		cg21099332	-	-	-	0.004	-	-	0.001
Plusquin, 2017	NO_2	cg08120023	*C1orf203*	Body	-	-0.003	-	-	0.001
		cg18201392	*RNF2*	5UTR	-	-0.005	-	-	0.001
		cg04914283	*EPHB2*	Body	-	-0.005	-	-	0.001
		cg18164357	*C11orf67*	5UTR	South shelf	-0.009	-	-	0.002
		cg16205861	-	-	South shore	-0.004	-	-	0.001
		cg03870188	*MCF2L*	Body	North shelf	-0.004	-	-	0.001
		cg12790758	*MEIS2*	Body	-	-0.004	-	-	0.001
		cg20939320	*NCRNA00119*	Body	-	-0.006	-	-	0.001
		cg21156210	*RG9MTD2*	TSS1500	Island	0.010	-	-	0.002
		cg13420207	*CACNA2D1*	Body	-	-0.010	-	-	0.002
		cg22856765	*THAP1*	3UTR	-	-0.008	-	-	0.001
		cg13918628	*CD72*	3UTR	-	-0.012	-	-	0.002
Sayols-Baixeras, 2019	NO_2	cg24172570	*HIBADH*	-	-	0.000	-	-	0.002
		cg08973675	*SLC25A28*	-	-	-0.001	-	-	0.003
		cg12283362	*LONP1*	-	-	0.000	-	-	0.002
White, 2019	NO_2	cg06544185	*Intergenic*	-	-	0.003	-	-	-
		cg02607340	*ACHE*	-	-	-0.007	-	-	-
NOx									
Chi, 2022	NOx	cg11756214	*ZNF347*	5' UTR	-	0.078	0.050	0.106	-
Plusquin, 2017	NOx	cg05036212	-	-	North shore	0.004	-	-	0.001
		cg08509991	*COL17A1*	TSS200	-	0.053	-	-	0.010
		cg09487985	-	-	North Shelf	-0.023	-	-	0.004
		cg18059012	*PATL2*	TSS200	-	-0.027	-	-	0.005
		cg18351711	*ODZ3*	TSS1500	-	-0.032	-	-	0.006
		cg12232118	*TRIM15*	5' UTR	-	-0.030	-	-	0.005
		cg09499965	-	-	South Shelf	-0.025	-	-	0.004
PAH									
Liu, 2021	OH-PAHs	cg09235308	*PLEC1; MIR661*	Body; TSS1500; TSS200	-	0.478	-	-	0.079
		cg02251027	*SSC5D*	Body	-	-0.500	-	-	0.107
		cg03426194	*GTF3C1*	Body	-	-0.500	-	-	0.109
		cg25136408	*RABEP1*	Body	-	-0.477	-	-	0.100
		cg23719549	*ABCB8*	Body	-	-0.501	-	-	0.108
		cg15641998	*HSD17B11*	1st Exon; 5′UTR	-	0.459	-	-	0.099
		cg18147606	*GFRA2*	Nearest gene	-	-0.282	-	-	0.066
PCB									
Pittman, 2020	PCBs	cg14251777	*GIMAP8*	-	-	-0.006	-	-	-
		cg12803754	*OTULIN*	-	-	-0.014	-	-	-
		cg00475490	*PRSS23*	-	-	-0.022	-	-	-
		cg00941989	*SH3TC1*	-	-	0.010	-	-	-
		cg25153882	*CTTNBP2*	-	-	0.009	-	-	-
		cg04991747	*GALNT2*	-	-	0.011	-	-	-
		cg14251777	*GIMAP8*	-	-	-0.006	-	-	-
		cg00475490	*PRSS23*	-	-	-0.022	-	-	-
		cg18861197	*PTK2B*	-	-	-0.011	-	-	-
		cg21566642	*AC068134.2*	-	-	-0.038	-	-	-
		cg25506215	*CA9*	-	-	0.011	-	-	-

		cg06005913	*CEP97*	-	-	0.006	-	-	-
		cg18974766	*CPNE5*	-	-	0.005	-	-	-
		cg26266985	*LOC101928185*	-	-	0.011	-	-	-
		cg12803754	*OTULIN*	-	-	-0.013	-	-	-
		cg23235442	*PFDN4*	-	-	-0.003	-	-	-
		cg00475490	*PRSS23*	-	-	-0.022	-	-	-
		cg18861197	*PTK2B*	-	-	-0.011	-	-	-
		cg00941989	*SH3TC1*	-	-	0.010	-	-	-
		cg04991747	*GALNT2*	-	-	0.011	-	-	-
		cg24512644	*ST13*	-	-	0.014	-	-	-
		cg08435853	*CCDC166*	-	-	0.040	-	-	-
		cg04991747	*GALNT2*	-	-	0.009	-	-	-
		cg11821245	*LDHC*	-	-	0.042	-	-	-
		cg25153882	*CTTNBP2*	-	-	0.008	-	-	-
		cg04991747	*GALNT2*	-	-	0.011	-	-	-
		cg24512644	*ST13*	-	-	0.013	-	-	-
		cg09639964	*TIMM22*	-	-	0.028	-	-	-
Curtis, 2021	PCBs	cg26207239	*BIRC2*	-	-	-	-	-	-
		cg19181419	-	-	-	-	-	-	-
		cg11828654	*TMEM187*	-	-	-	-	-	-
		cg17536959	-	-	-	-	-	-	-
		cg01607258	*CCDC130*	-	-	-	-	-	-
		cg03064005	*LRP5*	-	-	-	-	-	-
		cg14585892	*LY6D*	-	-	-	-	-	-
		cg18498987	*MEF2C*	-	-	-	-	-	-
		cg16716035	*OCRL*	-	-	-	-	-	-
		cg09551072	*SLC1A7*	-	-	-	-	-	-
		cg17978283	*BAIAP2;*	-	-	-	-	-	-
		cg19017060	*DDX27*	-	-	-	-	-	-
		cg13500824	*LEKR1;*	-	-	-	-	-	-
		cg23660678	*TSC22D3;*	-	-	-	-	-	-
		cg06114546	*ZC3H18;*	-	-	-	-	-	-
		cg16770948	*ZMYM3;*	-	-	-	-	-	-
		cg13041595	-	-	-	-	-	-	-
		cg13081398	*RBMS3;*	-	-	-	-	-	-
		cg05194114	*CCNDBP1;*	-	-	-	-	-	-
		cg01273397	-	-	-	-	-	-	-
		cg13378297	*LIMS2;*	-	-	-	-	-	-
		cg19839277	*TAF8*	-	-	-	-	-	-
		cg00179576	*GLG1;*	-	-	-	-	-	-
		cg15428169	*CD6;*	-	-	-	-	-	-
Other									
Alhamdow, 2017	∑OH-fluorene	cg03636183	*F2RL3*	-	-	-4.200	-7.600	-0.800	-
		cg05575921	*AHRR*	-	-	-10.000	-16.000	5.100	-

OH-PAHs=10 mono-hydroxylated PAHs; PCBs=polychlorinated biphenyl;

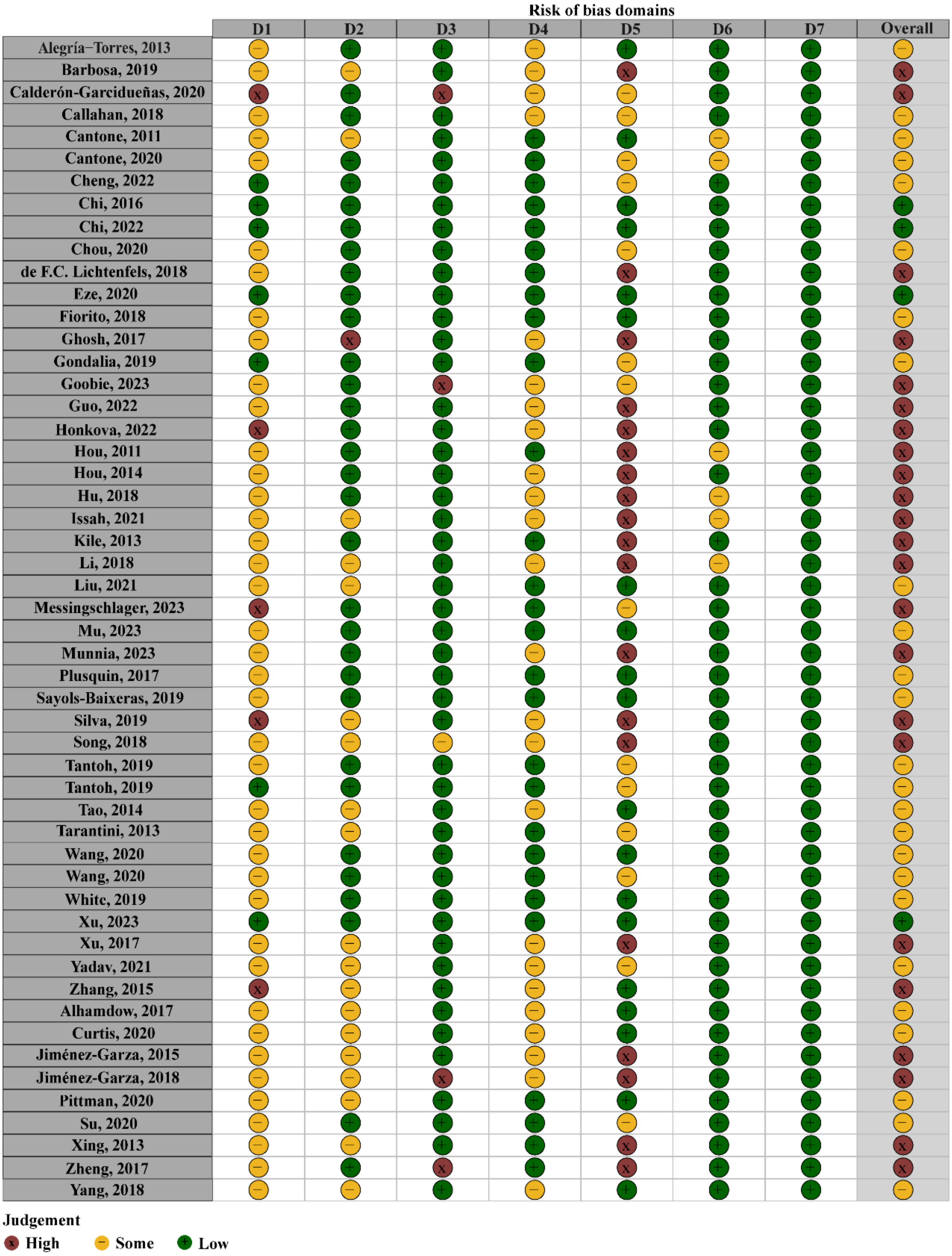

Figure S1. Traffic-light plots of the domain-level judgements for each study. D1: Bias due to confounding; D2: Bias due to exposure classification; D3: Bias due to missing data; D4: Bias arising from measurement of the outcome; D5: Bias arising from measurement of the exposure; D6: Bias due to reported results; D7: Bias due to selection of participants.

REFERENCES

1 Kile ML, Fang S, Baccarelli AA, Tarantini L, Cavallari J, Christiani DC. A panel study of occupational exposure to fine particulate matter and changes in DNA methylation over a single workday and years worked in boilermaker welders. Environ Health. 2013;12(1) (no pagination).

2 Munnia A, Bollati V, Russo V, Ferrari L, Ceppi M, Bruzzone M, et al. Traffic-Related Air Pollution and Ground-Level Ozone Associated Global DNA Hypomethylation and Bulky DNA Adduct Formation. Int J Mol Sci. 2023;24(3) (no pagination).

3 Yang J, Liu Y, Zhang H, Zhang H, Wang W, Fan Y. Urinary 1-hydroxypyrene and smoking are determinants of LINE-1 and AhRR promoter methylation in coke oven workers. Mutat Res Genet Toxicol Environ Mutagen. 2018;826:33-40.

4 Alegria-Torres JA, Barretta F, Batres-Esquivel LE, Carrizales-Yanez L, Perez-Maldonado IN, Baccarelli A, Bertazzi PA. Epigenetic markers of exposure to polycyclic aromatic hydrocarbons in Mexican brickmakers: a pilot study. Chemosphere. 2013;91:475-80.

5 Issah I, Arko-Mensah J, Rozek LS, Rentschler K, Agyekum TP, Dwumoh D, et al. Association between global DNA methylation (LINE-1) and occupational particulate matter exposure among informal electronic-waste recyclers in Ghana. Int J Environ Health Res. 2022;32:2406-24.

6 Wang C, O'Brien KM, Xu Z, Sandler DP, Taylor JA, Weinberg CR. Long-term ambient fine particulate matter and DNA methylation in inflammation pathways: results from the Sister Study. Epigenetics. 2020;15(5):524-35.

7 Guo L, Wang Y, Yang X, Wang T, Yin J, Zhao L, et al. Aberrant mitochondrial DNA methylation and declined pulmonary function in a population with polycyclic aromatic hydrocarbon composition in particulate matter. Environ Res. 2022;Part 1. 214 (no pagination).

8 Hou L, Zhang X, Zheng Y, Wang S, Dou C, Guo L, et al. Altered methylation in tandem repeat element and elemental component levels in inhalable air particles. Environ Mol Mutagen. 2014;55(3):256-65.

9 Xu Y, Li H, Hedmer M, Hossain MB, Tinnerberg H, Broberg K, Albin M. Occupational exposure to particles and mitochondrial DNA - relevance for blood pressure. Environ Health. 2017;16:22.

10 Alhamdow A, Essig YJ, Krais AM, Gustavsson P, Tinnerberg H, Lindh CH, et al. Fluorene exposure among PAH-exposed workers is associated with epigenetic markers related to lung cancer. Occup Environ Med. 2020;77:488-95.

11 Tarantini L, Bonzini M, Tripodi A, Angelici L, Nordio F, Cantone L, et al. Blood hypomethylation of inflammatory genes mediates the effects of metal-rich airborne pollutants on blood coagulation. Occup Environ Med. 2013;70(6):418-25.

12 Silva IR, Ramos M, Arantes L, Lengert AVH, Oliveira MA, Cury FP, et al. Evaluation of DNA Methylation Changes and Micronuclei in Workers Exposed to a Construction Environment. International Journal of Environmental Research & Public Health [Electronic Resource]. 2019;16:13.

13 Jiménez-Garza O, Baccarelli AA, Byun HM, Márquez-Gamiño S, Barrón-Vivanco BS, Albores A. CYP2E1 epigenetic regulation in chronic, low-level toluene exposure: Relationship with oxidative stress and smoking habit. Toxicol Appl Pharmacol. 2015;286:207-15.

14 Jiménez-Garza O, Guo L, Byun HM, Carrieri M, Bartolucci GB, Barrón-Vivanco BS, Baccarelli AA. Aberrant promoter methylation in genes related to hematopoietic malignancy in workers exposed to a VOC mixture. Toxicol Appl Pharmacol. 2018;339:65-72.
15 Callahan CL, Bonner MR, Nie J, Han D, Wang Y, Tao MH, et al. Lifetime exposure to ambient air pollution and methylation of tumor suppressor genes in breast tumors. Environ Res. 2018;161:418-24.
16 Cantone L, Tobaldini E, Favero C, Albetti B, Sacco RM, Torgano G, et al. Particulate air pollution, clock gene methylation, and stroke: Effects on stroke severity and disability. Int J Mol Sci. 2020;21(9) (no pagination).
17 Hou L, Zhang X, Tarantini L, Nordio F, Bonzini M, Angelici L, et al. Ambient PM exposure and DNA methylation in tumor suppressor genes: a cross-sectional study. Particle & Fibre Toxicology [Electronic Resource]. 2011;8:25.
18 Chi GC, Liu Y, MacDonald JW, Barr RG, Donohue KM, Hensley MD, et al. Long-term outdoor air pollution and DNA methylation in circulating monocytes: Results from the Multi-Ethnic Study of Atherosclerosis (MESA). Environ Health. 2016;15(1) (no pagination).
19 Plusquin M, Guida F, Polidoro S, Vermeulen R, Raaschou-Nielsen O, Campanella G, et al. DNA methylation and exposure to ambient air pollution in two prospective cohorts. Environ Int. 2017;108:127-36.
20 Xu R, Li S, Wu Y, Yue X, Wong EM, Southey MC, et al. Wildfire-related PM_{2.5} and DNA methylation: An Australian twin and family study. Environ Int. 2023;171:107704.
21 Chi GC, Liu Y, MacDonald JW, Reynolds LM, Enquobahrie DA, Fitzpatrick AL, et al. Epigenome-wide analysis of long-term air pollution exposure and DNA methylation in monocytes: results from the Multi-Ethnic Study of Atherosclerosis. Epigenetics: Official Journal of the DNA Methylation Society. 2022;17:297-313.
22 de FCLAJ, van der Plaat DA, de Jong K, van Diemen CC, Postma DS, Nedeljkovic I, et al. Long-term Air Pollution Exposure, Genome-wide DNA Methylation and Lung Function in the LifeLines Cohort Study. Environ Health Perspect. 2018;126:027004.
23 Eze IC, Jeong A, Schaffner E, Rezwan FI, Ghantous A, Foraster M, et al. Genome-wide DNA methylation in peripheral blood and long-term exposure to source-specific transportation noise and air pollution: The SAPALDIA study. Environ Health Perspect. 2020;128(6) (no pagination).
24 Fiorito G, Vlaanderen J, Polidoro S, Gulliver J, Galassi C, Ranzi A, et al. Oxidative stress and inflammation mediate the effect of air pollution on cardio- and cerebrovascular disease: A prospective study in nonsmokers. Environ Mol Mutagen. 2018;59(3):234-46.
25 Gondalia R, Baldassari A, Holliday KM, Justice AE, Mendez-Giraldez R, Stewart JD, et al. Methylome-wide association study provides evidence of particulate matter air pollution-associated DNA methylation. Environ Int. 2019;132 (no pagination).
26 Liu K, Jiang J, Lin Y, Liu W, Zhu X, Zhang Y, et al. Exposure to polycyclic aromatic hydrocarbons, DNA methylation and heart rate variability among non-current smokers. Environmental Pollution. 2021;288 (no pagination).

27 Sayols-Baixeras S, Fernandez-Sanles A, Prats-Uribe A, Subirana I, Plusquin M, Kunzli N, et al. Association between long-term air pollution exposure and DNA methylation: The REGICOR study. Environ Res. 2019;176 (no pagination).
28 White AJ, Kresovich JK, Keller JP, Xu Z, Kaufman JD, Weinberg CR, et al. Air pollution, particulate matter composition and methylation-based biologic age. Environ Int. 2019;132 (no pagination).
29 Chou YH, Tantoh DM, Wu MC, Tyan YS, Chen PH, Nfor ON, et al. PM<inf>2.5</inf> exposure and DLEC1 promoter methylation in Taiwan Biobank participants. Environ Health Prev Med. 2020;25(1) (no pagination).
30 Tantoh DM, Lee KJ, Nfor ON, Liaw YC, Lin C, Chu HW, et al. Methylation at cg05575921 of a smoking-related gene (AHRR) in non-smoking Taiwanese adults residing in areas with different PM_{2.5} concentrations. Clin Epigenetics. 2019;11:69.
31 Tantoh DM, Wu MF, Ho CC, Lung CC, Lee KJ, Nfor ON, et al. SOX2 promoter hypermethylation in non-smoking Taiwanese adults residing in air pollution areas. Clin Epigenetics. 2019;11(1) (no pagination).
32 Su CL, Tantoh DM, Chou YH, Wang L, Ho CC, Chen PH, et al. Blood-Based SOX2-Promoter Methylation in Relation to Exercise and PM(2.5) Exposure among Taiwanese Adults. Cancers (Basel). 2020;12.
33 Honkova K, Rossnerova A, Chvojkova I, Milcova A, Margaryan H, Pastorkova A, et al. Genome-Wide DNA Methylation in Policemen Working in Cities Differing by Major Sources of Air Pollution. Int J Mol Sci. 2022;23(3) (no pagination).
34 Mu G, Nie X, Yang S, Ye Z, Cheng M, Fan L, et al. PM_{2.5}-related DNA methylation and the association with lung function in non-smokers. Environmental Pollution. 2023;316:120700.
35 Curtis SW, Cobb DO, Kilaru V, Terrell ML, Marder ME, Barr DB, et al. Genome-wide DNA methylation differences and polychlorinated biphenyl (PCB) exposure in a US population. Epigenetics. 2021;16:338-52.
36 Pittman GS, Wang X, Campbell MR, Coulter SJ, Olson JR, Pavuk M, et al. Polychlorinated biphenyl exposure and DNA methylation in the Anniston Community Health Survey. Epigenetics. 2020;15:337-57.
37 Messingschlager M, Bartel-Steinbach M, Mackowiak SD, Denkena J, Bieg M, Klos M, et al. Genome-wide DNA methylation sequencing identifies epigenetic perturbations in the upper airways under long-term exposure to moderate levels of ambient air pollution. Environ Res. 2023;233 (no pagination).
38 Wang M, Zhao J, Wang Y, Mao Y, Zhao X, Huang P, et al. Genome-wide DNA methylation analysis reveals significant impact of long-term ambient air pollution exposure on biological functions related to mitochondria and immune response. Environmental Pollution. 2020;264 (no pagination).
39 Tao MH, Zhou J, Rialdi AP, Martinez R, Dabek J, Scelo G, et al. Indoor air pollution from solid fuels and peripheral Blood DNA methylation: Findings from a population study in Warsaw, Poland. Environ Res. 2014;134:325-30.

40 Cheng Y, Tang Q, Lu Y, Li M, Zhou Y, Wu P, et al. Semen quality and sperm DNA methylation in relation to long-term exposure to air pollution in fertile men: A cross-sectional study. Environmental Pollution. 2022;300 (no pagination).

41 Goobie GC, Li X, Ryerson CJ, Carlsten C, Johannson KA, Fabisiak JP, et al. PM<inf>2.5</inf> and constituent component impacts on global DNA methylation in patients with idiopathic pulmonary fibrosis. Environmental pollution (Barking, Essex : 1987). 2022:120942.

42 Yadav S, Longkumer I, Garg PR, Joshi S, Rajkumari S, Devi NK, Saraswathy KN. Association of air pollution and homocysteine with global DNA methylation: A population-based study from North India. PLoS One. 2021;16(12 December) (no pagination).

43 Cantone L, Nordio F, Hou L, Apostoli P, Bonzini M, Tarantini L, et al. Inhalable metal-rich air particles and histone H3K4 dimethylation and H3K9 Acetylation in a Cross-sectional Study of Steel Workers. Environ Health Perspect. 2011;119(7):964-9.

44 Li J, Xing X, Zhang X, Liang B, He Z, Gao C, et al. Enhanced H3K4me3 modifications are involved in the transactivation of DNA damage responsive genes in workers exposed to low-level benzene. Environmental Pollution. 2018;234:127-35.

45 Song X, Miao M, Zhou X, Li D, Tian Y, Liang H, et al. Bisphenol A Exposure and Sperm ACHE Hydroxymethylation in Men. International Journal of Environmental Research & Public Health [Electronic Resource]. 2019;16:08.

46 Zhang H, Li X, Ge L, Yang J, Sun J, Niu Q. Methylation of CpG island of p14(ARK), p15(INK4b) and p16(INK4a) genes in coke oven workers. Hum Exp Toxicol. 2015;34:191-7.

47 Barbosa E, Dos Santos ALA, Peteffi GP, Schneider A, Muller D, Rovaris D, et al. Increase of global DNA methylation patterns in beauty salon workers exposed to low levels of formaldehyde. Environ Sci Pollut Res Int. 2019;26(2):1304-14.

48 Ghosh M, Oner D, Poels K, Tabish AM, Vlaanderen J, Pronk A, et al. Changes in DNA methylation induced by multi-walled carbon nanotube exposure in the workplace. Nanotoxicology. 2017;11:1195-210.

49 Hu G, Li P, Cui X, Li Y, Zhang J, Zhai X, et al. Cr(VI)-induced methylation and down-regulation of DNA repair genes and its association with markers of genetic damage in workers and 16HBE cells. Environmental Pollution. 2018;238:833-43.

50 Xing C, Chen Q, Li G, Zhang L, Zheng M, Zou Z, et al. Microsomal epoxide hydrolase (EPHX1) polymorphisms are associated with aberrant promoter methylation of ERCC3 and hematotoxicity in benzene-exposed workers. Environ Mol Mutagen. 2013;54:397-405.

51 Zheng M, Lin F, Hou F, Li G, Zhu C, Xu P, et al. Association between Promoter Methylation of Gene ERCC3 and Benzene Hematotoxicity. Int J Environ Res Public Health. 2017;14.

52 Calderon-Garciduenas L, Herrera-Soto A, Jury N, Maher BA, Gonzalez-Maciel A, Reynoso-Robles R, et al. Reduced repressive epigenetic marks, increased DNA damage and Alzheimer's disease hallmarks in the brain of humans and mice exposed to particulate urban air pollution. Environ Res. 2020;183 (no pagination).

PRISMA 2020 Checklist

Section and Topic	Item #	Checklist item	Location where item is reported
**TITLE**			
Title	1	Identify the report as a systematic review.	2
**ABSTRACT**			
Abstract	2	See the PRISMA 2020 for Abstracts checklist.	2
**INTRODUCTION**			
Rationale	3	Describe the rationale for the review in the context of existing knowledge.	3
Objectives	4	Provide an explicit statement of the objective(s) or question(s) the review addresses.	4
**METHODS**			
Eligibility criteria	5	Specify the inclusion and exclusion criteria for the review and how studies were grouped for the syntheses.	4, 5
Information sources	6	Specify all databases, registers, websites, organisations, reference lists and other sources searched or consulted to identify studies. Specify the date when each source was last searched or consulted.	4
Search strategy	7	Present the full search strategies for all databases, registers and websites, including any filters and limits used.	4
Selection process	8	Specify the methods used to decide whether a study met the inclusion criteria of the review, including how many reviewers screened each record and each report retrieved, whether they worked independently, and if applicable, details of automation tools used in the process.	4, 5
Data collection process	9	Specify the methods used to collect data from reports, including how many reviewers collected data from each report, whether they worked independently, any processes for obtaining or confirming data from study investigators, and if applicable, details of automation tools used in the process.	5
Data items	10a	List and define all outcomes for which data were sought. Specify whether all results that were compatible with each outcome domain in each study were sought (e.g. for all measures, time points, analyses), and if not, the methods used to decide which results to collect.	5
	10b	List and define all other variables for which data were sought (e.g. participant and intervention characteristics, funding sources). Describe any assumptions made about any missing or unclear information.	5
Study risk of bias assessment	11	Specify the methods used to assess risk of bias in the included studies, including details of the tool(s) used, how many reviewers assessed each study and whether they worked independently, and if applicable, details of automation tools used in the process.	5, 6
Effect measures	12	Specify for each outcome the effect measure(s) (e.g. risk ratio, mean difference) used in the synthesis or presentation of results.	5
Synthesis methods	13a	Describe the processes used to decide which studies were eligible for each synthesis (e.g. tabulating the study intervention characteristics and comparing against the planned groups for each synthesis (item #5)).	4, 5

Section and Topic	Item #	Checklist item	Location where item is reported
	13b	Describe any methods required to prepare the data for presentation or synthesis, such as handling of missing summary statistics, or data conversions.	5
	13c	Describe any methods used to tabulate or visually display results of individual studies and syntheses.	5, 6
	13d	Describe any methods used to synthesize results and provide a rationale for the choice(s). If meta-analysis was performed, describe the model(s), method(s) to identify the presence and extent of statistical heterogeneity, and software package(s) used.	4-6
	13e	Describe any methods used to explore possible causes of heterogeneity among study results (e.g. subgroup analysis, meta-regression).	5, 6
	13f	Describe any sensitivity analyses conducted to assess robustness of the synthesized results.	Not applicable
Reporting bias assessment	14	Describe any methods used to assess risk of bias due to missing results in a synthesis (arising from reporting biases).	5
Certainty assessment	15	Describe any methods used to assess certainty (or confidence) in the body of evidence for an outcome.	6
**RESULTS**			
Study selection	16a	Describe the results of the search and selection process, from the number of records identified in the search to the number of studies included in the review, ideally using a flow diagram.	6, 7
	16b	Cite studies that might appear to meet the inclusion criteria, but which were excluded, and explain why they were excluded.	6
Study characteristics	17	Cite each included study and present its characteristics.	6, 7
Risk of bias in studies	18	Present assessments of risk of bias for each included study.	7, 8
Results of individual studies	19	For all outcomes, present, for each study: (a) summary statistics for each group (where appropriate) and (b) an effect estimate and its precision (e.g. confidence/credible interval), ideally using structured tables or plots.	9-11
cResults of syntheses	20a	For each synthesis, briefly summarise the characteristics and risk of bias among contributing studies.	7, 8
	20b	Present results of all statistical syntheses conducted. If meta-analysis was done, present for each the summary estimate and its precision (e.g. confidence/credible interval) and measures of statistical heterogeneity. If comparing groups, describe the direction of the effect.	Not applicable
	20c	Present results of all investigations of possible causes of heterogeneity among study results.	7, 8
	20d	Present results of all sensitivity analyses conducted to assess the robustness of the synthesized results.	Not applicable
Reporting biases	21	Present assessments of risk of bias due to missing results (arising from reporting biases) for each synthesis assessed.	7

Section and Topic	Item #	Checklist item	Location where item is reported
Certainty of evidence	22	Present assessments of certainty (or confidence) in the body of evidence for each outcome assessed.	7, 8
**DISCUSSION**			
Discussion	23a	Provide a general interpretation of the results in the context of other evidence.	11, 12
	23b	Discuss any limitations of the evidence included in the review.	14, 15
	23c	Discuss any limitations of the review processes used.	16
	23d	Discuss implications of the results for practice, policy, and future research.	16
**OTHER INFORMATION**			
Registration and protocol	24a	Provide registration information for the review, including register name and registration number, or state that the review was not registered.	2, 4
	24b	Indicate where the review protocol can be accessed, or state that a protocol was not prepared.	2, 4
	24c	Describe and explain any amendments to information provided at registration or in the protocol.	2, 4
Support	25	Describe sources of financial or non-financial support for the review, and the role of the funders or sponsors in the review.	16
Competing interests	26	Declare any competing interests of review authors.	17
Availability of data, code and other materials	27	Report which of the following are publicly available and where they can be found: template data collection forms; data extracted from included studies; data used for all analyses; analytic code; any other materials used in the review.	16

From: Page MJ, McKenzie JE, Bossuyt PM, Boutron I, Hoffmann TC, Mulrow CD, et al. The PRISMA 2020 statement: an updated guideline for reporting systematic reviews. BMJ 2021;372:n71. doi: 10.1136/bmj.n71. This work is licensed under CC BY 4.0. To view a copy of this license, visit https://creativecommons.org/licenses/by/4.0/
